# Albumin-Binding Moieties Tune the Pharmacokinetics and Tumor Uptake of ^177^Lu-Labeled GRPR Radioligands

**DOI:** 10.1021/acsmedchemlett.6c00338

**Published:** 2026-09-04

**Authors:** Karim Obeid, Esther Olaniran Håkansson, Ekaterina Bezverkhniaia, Bobo Skillinghaug, Ulrika Rosenström, Panagiotis Kanellopoulos, Anna Orlova, Luke R. Odell

**Affiliations:** † Department of Medicinal Chemistry, 8097 BMC Uppsala University, P.O. Box 574, SE-751 23 Uppsala, Sweden; ‡ Science for Life Laboratory, Uppsala University, 752 37 Uppsala, Sweden

**Keywords:** Gastrin-releasing peptide receptor, albumin-binding moiety, radiotheranostics

## Abstract

Although the gastrin-releasing peptide receptor (GRPR) is an established theranostic target across multiple cancers, many GRPR-targeting peptides undergo rapid renal clearance, limiting tumor uptake and therapeutic exposure. Albumin-binding moieties (ABMs) reversibly interact with serum albumin, prolonging systemic exposure and increasing the opportunity for tumor uptake. To examine how ABM structure and placement affect this behavior, we prepared four DOTA-functionalized GRPR-targeting conjugates, varying both ABM lipophilicity and position relative to the DOTA chelator. The conjugates were radiolabeled with lutetium-177 in radiochemical yields and purities of >98%. All radioconjugates retained GRPR-mediated uptake in PC-3 cells, exhibited subnanomolar receptor affinity and enhanced blood retention. [^177^Lu]­Lu-CA6356, bearing a 4-(*p*-iodophenyl)­butyric acid-derived ABM, achieved the highest tumor uptake in PC-3 xenografts at 4 h pi (12 ± 2% IA/g), which increased by approximately 30% 24 h pi. These findings show that ABM identity and placement can modulate systemic exposure and tumor uptake in GRPR-targeting radioligands.

## Graphical abstract

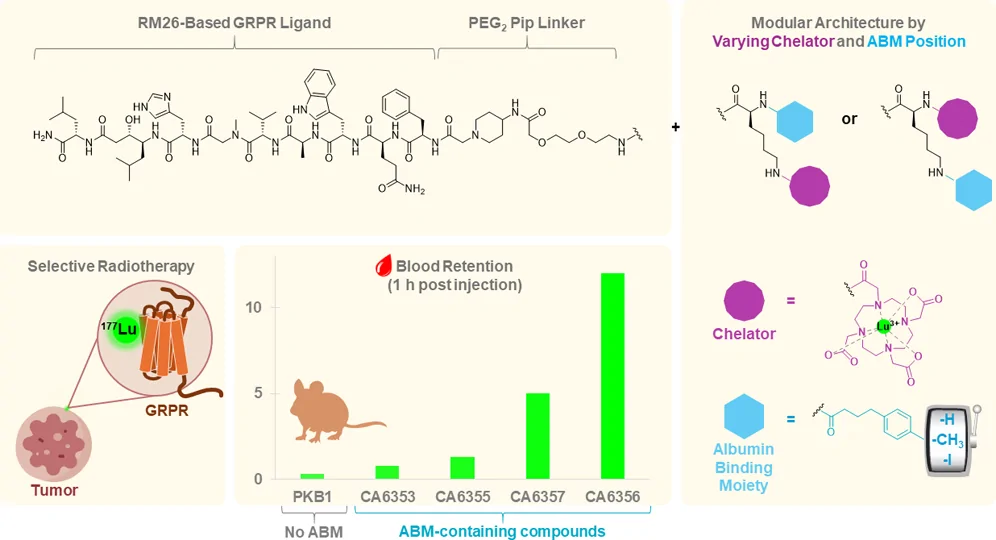

The gastrin-releasing peptide receptor (GRPR) is overexpressed in several tumor types such as prostate, breast, gastric, pancreatic, colorectal and small cell lung carcinomas, making it an attractive molecular target in oncology.^1^ Gastrin-releasing peptide, the endogenous ligand of GRPR and the related amphibian peptide bombesin (BBN) have inspired the development of several GRPR-targeting radiopharmaceuticals, such as DB1, DB15, SB3, NeoBOMB1, RM2 and NOTA-PEG_2_-RM26.^2^ Antagonistic GRPR radioligands are generally preferred over agonists for radiopharmaceutical development because they avoid agonist-induced receptor activation and associated side effects. Despite low internalization, antagonists can achieve high tumor uptake and rapid clearance from healthy tissues, including GRPR-expressing organs such as the pancreas, resulting in favorable pharmacokinetic profiles.^2,3^

GRPR-targeting radioligands have demonstrated strong potential in theranostic applications, where structurally similar compounds are labeled with different radioisotopes for both diagnostic imaging (e.g., gallium-68) and therapeutic applications (e.g., lutetium-177).^4–6^ However, peptide-based pharmaceuticals are often limited by enzymatic degradation and clearance, reducing the amount of intact radioligand available for receptor-mediated targeting. BBN-based GRPR ligands are particularly susceptible to degradation by neprilysin (NEP), resulting in reduced tumor uptake.^2^ Previous work from our group addressed this limitation by modifying the RM26 scaffold to improve resistance to enzymatic degradation.^8^ This effort led to the development of [^111^In]­In-AU-RM26-M2, an analogue of DOTAGA-PEG_2_-RM26,^7^ in which Gly^11^ was replaced with Sar^11^ (*N*-methylglycine) and the PEG_2_ linker was extended with a positively charged piperidine. Although [^111^In]­In-AU-RM26-M2 exhibited improved resistance to NEP degradation and rapid clearance from nontarget tissues, its increased renal uptake and limited systemic exposure remained potential obstacles for therapeutic application.

A promising strategy to overcome this challenge involves leveraging human serum albumin (HSA), a large (ca. 66.5 kDa), highly abundant plasma protein (35–50 g/L) with a long *in vivo* half-life (ca. 19 days).^9,10^ HSA acts as a transporter for various endogenous and exogenous compounds, including fatty acids and drug molecules. Noncovalent association with HSA increases the effective molecular size of radioligands, which extends systemic circulation, reduces renal excretion and increases blood exposure. Fusion with a large albumin-binding domain (ABD, 5 kDa) from Streptococcal protein G has been shown to prolong half-life and enhance tumor uptake.^11^ However, the size of these scaffolds may limit receptor binding, restrict tissue penetration and introduce synthetic challenges.

An alternative approach involves the use of small-molecule albumin-binding moieties (ABMs), such as fatty acid or butyric acid derivatives, which can be more readily incorporated into peptide scaffolds than large protein-based domains. In 2008, screening of a DNA-encoded library identified 4-phenylbutanoic acid derivatives as a new class of ABMs, with 4-(*p*-iodophenyl)­butanoic acid showing the highest albumin affinity.^12^ This motif has since been incorporated into several peptide radioligands, including radioconjugates targeting prostate-specific membrane antigen and GRPR.^13–18^

In this study, we investigated how ABM identity and positioning influence GRPR binding and the pharmacokinetic behavior of GRPR-targeting radioligands. We designed four novel PKB1^19^-based conjugates bearing two structurally related ABMs, derived from 4-(*p*-iodophenyl)­butyric acid (ABM1) and 4-(*p*-tolyl)­butyric acid (ABM2). These ABMs were selected based on established albumin-binding affinity and distinct physicochemical properties.^12,20^ To independently evaluate the effects of ABM lipophilicity and conjugate architecture, the ABM and DOTA chelator were positioned in alternative arrangements around an orthogonally functionalized lysine branching point. This design enabled systematic assessment of how ABM lipophilicity and placement modulate blood retention and tissue distribution through altered albumin interactions. All conjugates incorporated an RM26-based GRPR peptide containing the Gly^11^/Sar^11^ substitution, together with a PEG_2_-piperidine linker and a DOTA chelator for radiometal complexation.

To translate this modular design into the target conjugates, a route-divergent solid-phase peptide synthesis (SPPS) strategy was employed, centered on an orthogonally protected N-terminal lysine residue. Fmoc protection of the α-amino group and Alloc protection of the lysine side-chain amine allowed selective deprotection of either site, enabling divergent installation of the ABM and DOTA chelator (Scheme 1). In contrast to previously reported GRPR conjugates such as AMTG-DA1 and L24, where the ABM was incorporated within a fixed structural arrangement,^16^ this design enables systematic evaluation of how ABM placement influences biological behavior.

**Scheme 1:**
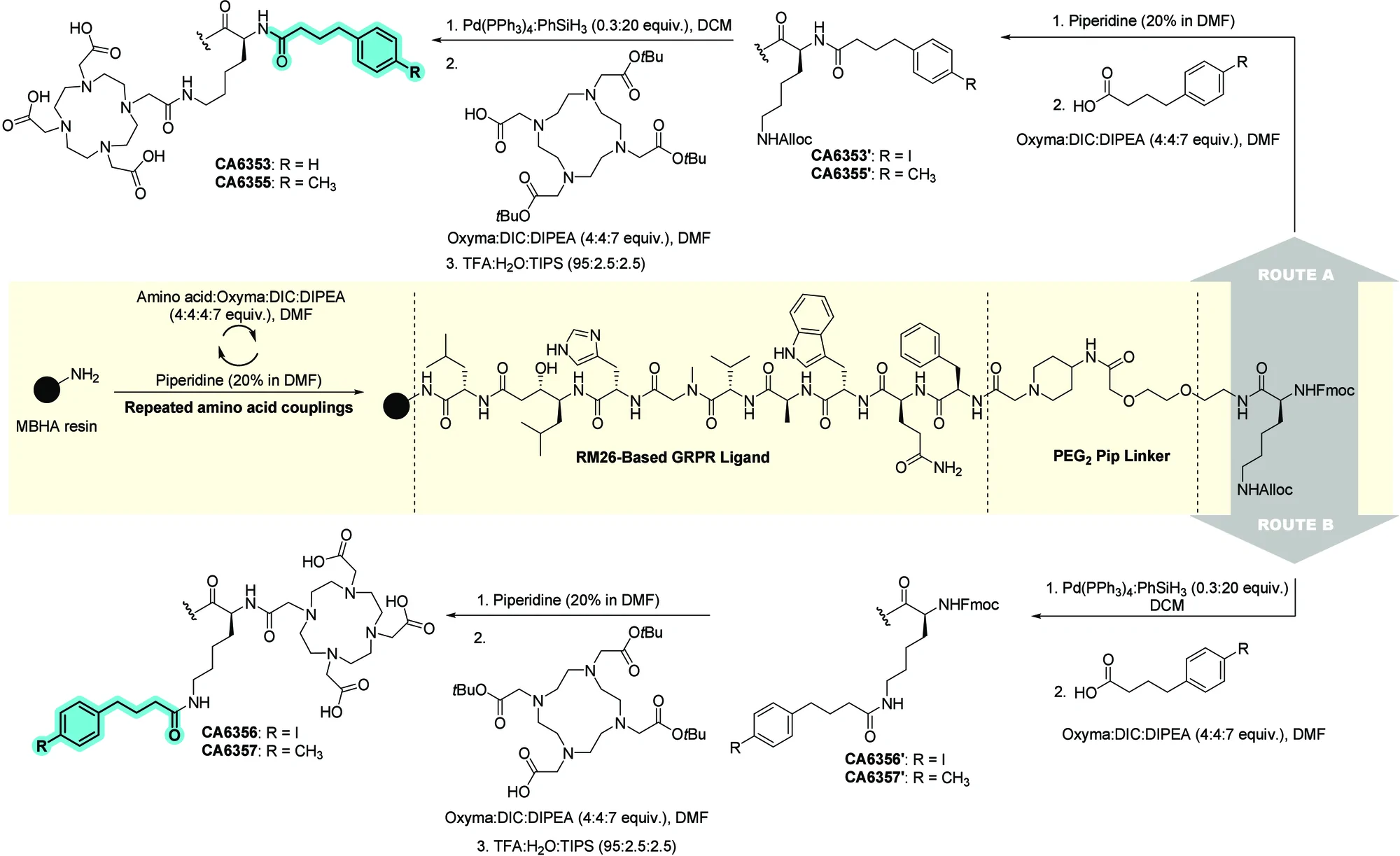
Synthetic Strategy for Preparation of Novel GRPR Conjugates via SPPS^a^

a Orthogonal deprotection of Fmoc and Alloc enabled alternative placement of ABM and DOTA around a lysine branching point.

All new conjugates were synthesized by SPPS using iterative Oxyma/DIC-mediated coupling and N-terminal Fmoc deprotection cycles. Selective Alloc removal from the lysine side-chain was performed using Pd­(PPh_3_)_4_ and phenylsilane in DCM. For N-terminal ABM placement, the ABMs were coupled prior to palladium-catalyzed Alloc deprotection of the side-chain and DOTA conjugation (Scheme 1). However, the iodinated intermediate underwent complete dehalogenation under these conditions, yielding phenylbutyric acid as the ABM (conjugate CA6353). Although phenylbutyric acid has weaker albumin affinity than more lipophilic analogues,^10^ CA6353 provides a useful comparison to CA6355 due to their differing lipophilicity.

For CA6356 and CA6357, the DOTA chelator was attached to the lysine side-chain following palladium-catalyzed Alloc deprotection, with subsequent coupling of ABM1 and ABM2 under standard conditions (Scheme 1). Finally, global deprotection and cleavage from resin under acidic conditions afforded the crude peptides, which were purified by reversed-phase high-performance liquid chromatography (RP-HPLC) to yield the final products as white solids in 7–9% overall yield (Figures S1–S8).

Radiolabeling of the peptides with Lu-177 was performed by incubation with [^177^Lu]­LuCl_3_ in ammonium acetate buffer (0.2 M, pH 5.5) and EtOH (99.5%) at 85 °C for 30 min. [^177^Lu]­Lu-CA6353, [^177^Lu]­Lu-CA6355, [^177^Lu]­Lu-CA6356 and [^177^Lu]­Lu-CA6357 were obtained with radiochemical yields and purities exceeding 98% (Table 1 and Figures S9–S12). Initial physicochemical characterization (LogD) identified [^177^Lu]­Lu-CA6356 as the most lipophilic compound, followed by [^177^Lu]­Lu-CA6357, [^177^Lu]­Lu-CA6353 and [^177^Lu]­Lu-CA6355 (Table 1). Notably, CA6355 and CA6357, which incorporate the same ABM but differ in the site of conjugation, exhibited distinct lipophilicity, indicating that ABM positioning directly influences the effective physicochemical profile of the conjugates.

**Table 1:**
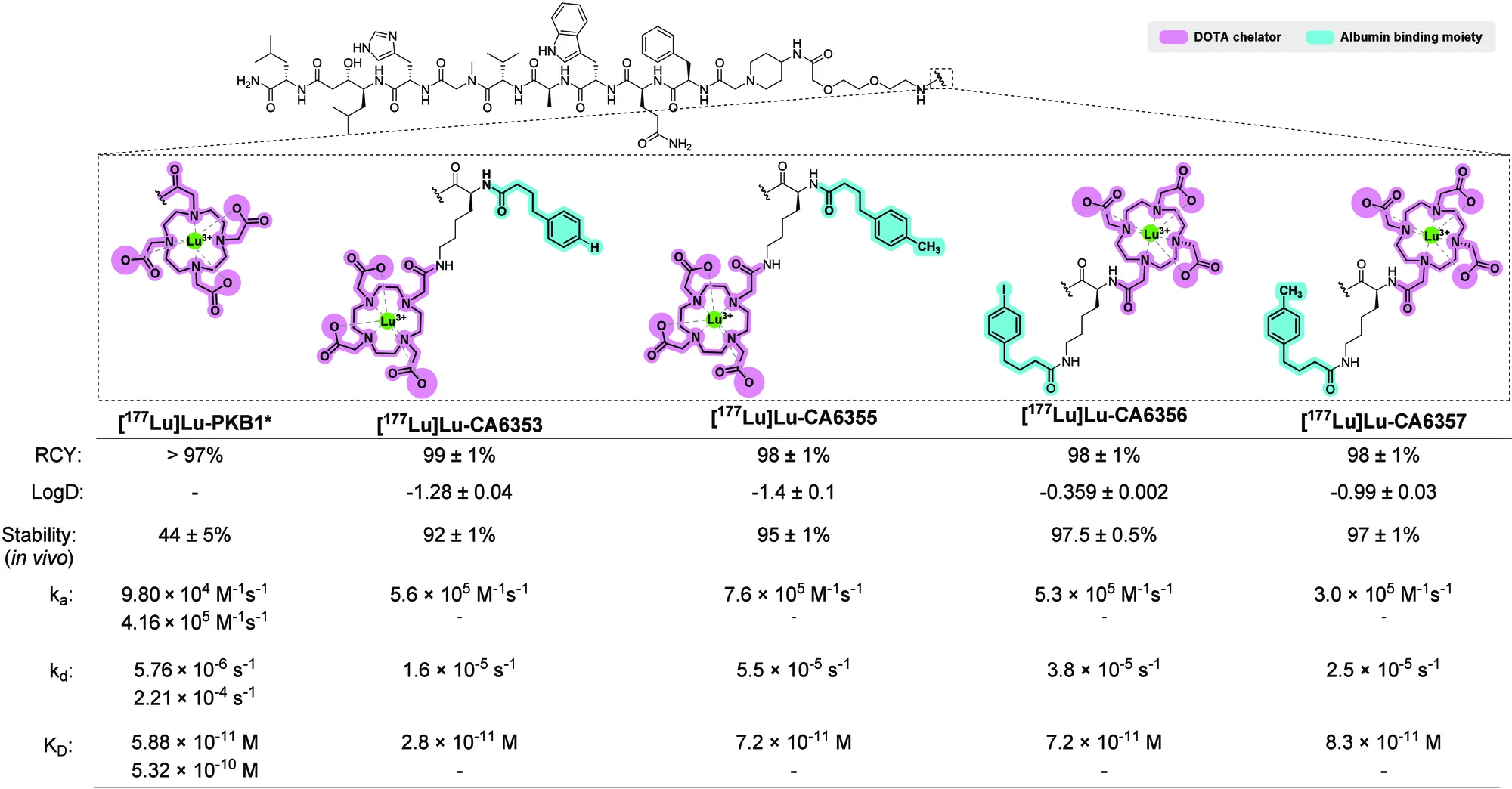
Structures and Characterization Data for the Comparator [^177^Lu]­Lu-PKB1* and the Novel Compounds [^177^Lu]­Lu-CA6353, [^177^Lu]­Lu-CA6355, [^177^Lu]­Lu-CA6356 and [^177^Lu]­Lu-CA6357^a^

a RCY = radiochemical yield, LogD = distribution coefficient, In vivo stability = % of intact peptide in blood plasma 5 min p.i., *k*_a_ = association constant, *k*_d_ = dissociation constant, K_D_ = equilibrium dissociation constant. *Data from Obeid et al. 2025.^19^

The new radioconjugates were first evaluated in vitro in GRPR-expressing PC-3 cells. To determine whether these properties translated in vivo, biodistribution studies were subsequently performed in healthy NMRI mice and Balb/c nu/nu mice bearing PC-3 xenografts. The peptide [^177^Lu]­Lu-PKB1 without ABM incorporation was used for comparison of pharmacological properties.^19^

Real-time affinity measurements on living PC-3 cells revealed that all compounds bind GRPR with subnanomolar affinity (K_D_ values, Table 1). Representative sensorgrams obtained with LigandTracer are provided in Figure S13. In contrast, [^177^Lu]­Lu-PKB1 exhibited two binding components, both in the subnanomolar range, suggesting that ABM incorporation restricts peptide flexibility. Importantly, neither ABM incorporation nor its position relative to the DOTA chelator compromised GRPR binding, in contrast to previously reported ABM- or ABD-modified conjugates that exhibited reduced affinity.^11,16^ Interestingly, [^177^Lu]­Lu-CA6353, the most hydrophilic conjugate, displayed the strongest affinity for GRPR, whereas lipophilic modifications are more typically associated with enhanced receptor binding.^21^

The in vitro binding specificity was confirmed by comparing peptide uptake in blocked (pretreated with excess unlabeled GRPR-ligand) and nonblocked cells. All radioconjugates displayed GRPR-mediated uptake, with a significant reduction under blocking conditions (*p* < 0.0001) (Figure 1A). Among the series, [^177^Lu]­Lu-CA6356, the most lipophilic conjugate, showed the highest GRPR-specific activity uptake. The series exhibited slow internalization, with approximately 20% of cell-associated activity internalized after 24 h (Figure 1B), consistent with the expected behavior of GRPR antagonists.^2^

**Figure 1:**
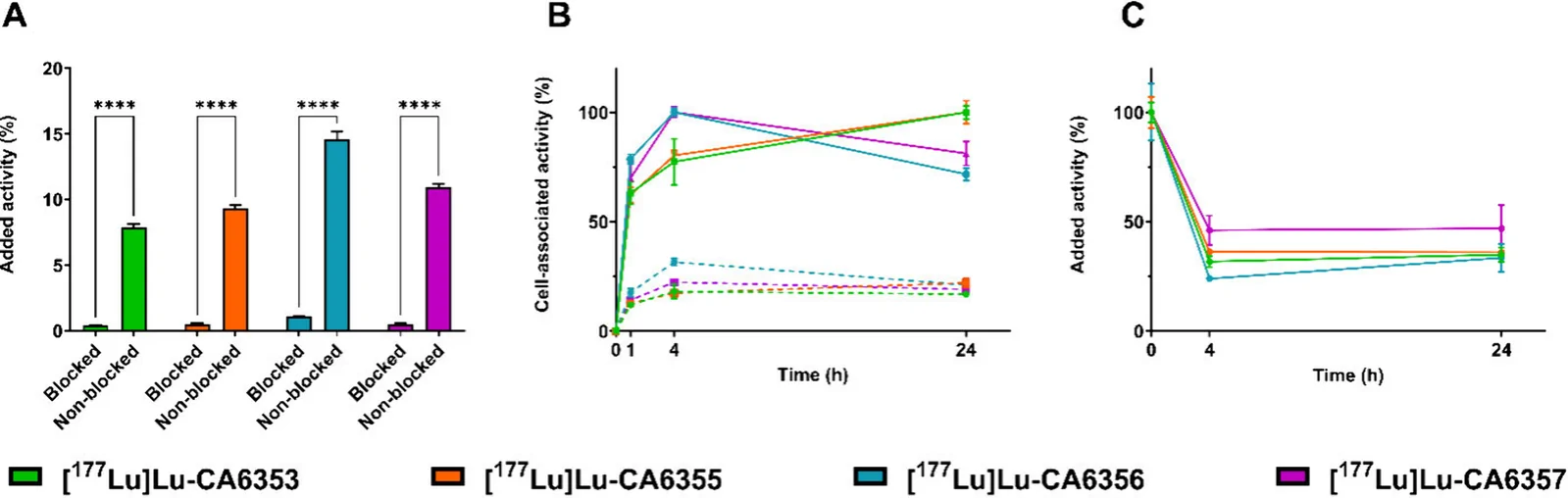
In vitro specificity assay (**A**), cellular processing with internalization under continuous incubation (**B**) and cellular retention (**C**) after interrupted incubation for [^177^Lu]­Lu-CA6353, [^177^Lu]­Lu-CA6355, [^177^Lu]­Lu-CA6356 and [^177^Lu]­Lu-CA6357 on PC-3 cells. **** indicates p value <0.0001. Full line: Total activity; Dashed line: Internalized fraction. Some error bars are not visible due to low variability.

Following receptor binding, we next examined cellular processing and retention. All conjugates exhibited a similar processing profile, characterized by rapid binding followed by slow internalization. Conjugates with the ABM on the side chain ([^177^Lu]­Lu-CA6356 and [^177^Lu]­Lu-CA6357) demonstrated significantly lower cell-associated activity at 24 h relative to 4 h, while N-terminally modified analogues continued to accumulate. This likely reflects differences in the residualizing properties of radiocatabolites, with [^177^Lu]­Lu-CA6356 and [^177^Lu]­Lu-CA6357 generating less residualizing species than [^177^Lu]­Lu-CA6353 and [^177^Lu]­Lu-CA6355.

Cellular retention was also assessed up to 24 h post GRPR saturation (Figure 1C). Retention remained largely constant between 4 and 24 h for all compounds. Notably, [^177^Lu]­Lu-CA6357 displayed the highest retention, with 46% of the added activity remaining at 24 h, whereas the other analogues retained no more than 35%.

Given the favorable in vitro profile of the series, we next assessed whether ABM incorporation affected metabolic stability in vivo. A metabolite assay was therefore performed in healthy NMRI mice (Figures S14–S18). At 5 min postinjection (pi), all conjugates showed high metabolic stability, with over 90% of activity detected in blood associated with intact radiopeptide (Table 1). Among these, [^177^Lu]­Lu-CA6356 and [^177^Lu]­Lu-CA6357 showed the highest stability (97% intact peptide). In contrast, the non-ABM analogue [^177^Lu]­Lu-PKB1 had only 44% of intact peptide at 5 min pi,^19^ indicating that ABM incorporation protects the conjugates from enzymatic degradation. Notably, even at later time points, [^177^Lu]­Lu-CA6356 maintained a high metabolic stability with 92% of intact radiopeptide detected at 1 h pi (Figure S18).

To assess how ABM composition and positioning influence pharmacokinetics, the biodistribution profiles of the conjugates were compared with [^177^Lu]­Lu-PKB1 in healthy NMRI mice (Figure 2 and Table S1). All ABM-containing conjugates exhibited significantly higher blood activity at 1 h pi than the reference peptide, indicating that ABM incorporation enhances systemic retention. This is consistent with previous reports for both small-molecule ABMs and ABD-based constructs.^11,15,16^

**Figure 2:**
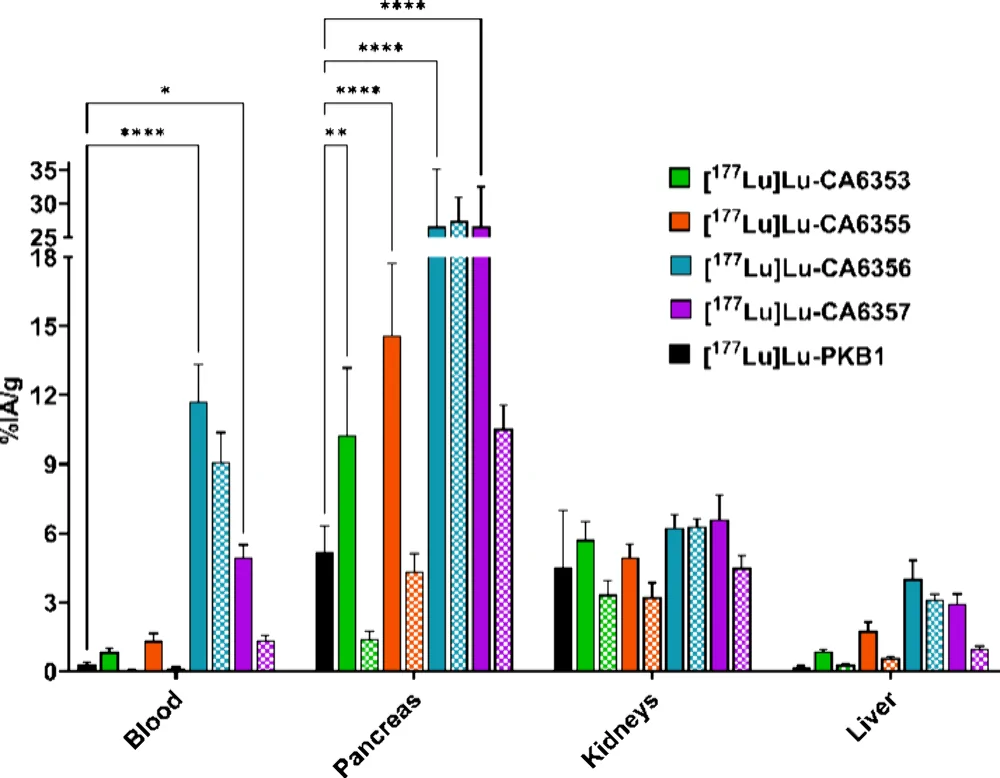
Biodistribution of [^177^Lu]­Lu-PKB1, [^177^Lu]­Lu-CA6353, [^177^Lu]­Lu-CA6355, [^177^Lu]­Lu-CA6356 and [^177^Lu]­Lu-CA6357 in healthy NMRI mice. Full pattern: 1 h pi; squared pattern: 4 h pi. Data are presented as mean ± SD (*n* = 4). Statistical differences are shown as * (*p* < 0.05), ** (*p* < 0.01) and **** (*p* < 0.0001).

Within the series, [^177^Lu]­Lu-CA6356 exhibited the highest blood activity concentration at 1 h pi, which was maintained at 4 h pi. In contrast, [^177^Lu]­Lu-CA6357 showed moderate blood levels at 1 h pi that decreased significantly at 4 h pi. [^177^Lu]­Lu-CA6353 and [^177^Lu]­Lu-CA6355 displayed substantially lower blood concentrations already at 1 h pi. As [^177^Lu]­Lu-CA6356 and [^177^Lu]­Lu-CA6357 differ only in the ABM substituent (iodo vs methyl), these differences likely reflect variation in albumin-binding strength and duration, consistent with their lipophilicity. Prolonged circulation of [^177^Lu]­Lu-CA6356 was further reflected in higher retention in both GRPR-expressing (pancreas) and nontarget tissues (kidneys and liver).

Furthermore, comparison of [^177^Lu]­Lu-CA6355 and [^177^Lu]­Lu-CA6357 shows that both incorporate the same ABM (4-(*p*-tolyl)­butyric acid), yet [^177^Lu]­Lu-CA6357, bearing side-chain attachment, showed a substantially higher blood retention and thus a more favorable pharmacokinetic profile. In contrast, [^177^Lu]­Lu-CA6355, with N-terminal placement, displayed lower blood levels and rapid washout from GRPR-expressing tissues. Pancreatic uptake peaked early but declined sharply at 4 h pi, consistent with more rapid systemic clearance, indicating that ABM placement influences pharmacokinetic behavior, potentially through altered presentation of the albumin-binding moiety and its interaction with circulating albumin. These findings demonstrate that ABM positioning alone is sufficient to modulate pharmacokinetic behavior, independently of ABM identity.

As a GRPR-expressing tissue, the pancreas provides a useful surrogate for receptor-mediated uptake.^22^ All radioconjugates demonstrated higher pancreatic uptake than [^177^Lu]­Lu-PKB1 at 1 h pi. [^177^Lu]­Lu-CA6353 and [^177^Lu]­Lu-CA6355 exhibited low uptake and rapid washout from the pancreas, which is typical for GRPR antagonists,^2^ whereas [^177^Lu]­Lu-CA6356 and [^177^Lu]­Lu-CA6357 showed the highest pancreatic uptake at 1 h pi. [^177^Lu]­Lu-CA6356 maintained elevated pancreatic levels at 4 h pi, while [^177^Lu]­Lu-CA6357 showed a moderate decline over the same period. Given that the pancreas is relatively radioresistant and is not typically dose-limiting in radionuclide therapy,^23^ high pancreatic uptake is less concerning for off-target toxicity and can serve as a proxy for GRPR-mediated tumor targeting. Accordingly, [^177^Lu]­Lu-CA6356 is expected to exhibit the highest tumor accumulation in vivo.

All compounds were primarily excreted through the kidneys and showed a similarly low renal uptake, comparable to [^177^Lu]­Lu-PKB1. As the most lipophilic analogue, [^177^Lu]­Lu-CA6356 also demonstrated the highest liver uptake at 1 h pi; however, all compounds showed low hepatic accumulation with no significant differences relative to [^177^Lu]­Lu-PKB1.

While prolonged systemic circulation is beneficial for tumor targeting, extended blood retention may also increase radiation exposure to nontarget tissues, particularly bone marrow, potentially reducing the therapeutic index. To determine whether differences in circulation time translated into tumor targeting, biodistribution studies were performed in PC-3 xenografted mice using three conjugates with different blood retention profiles: [^177^Lu]­Lu-CA6355 (low retention), [^177^Lu]­Lu-CA6357 (intermediate) and [^177^Lu]­Lu-CA6356 (high) in comparison with [^177^Lu]­Lu-PKB1 (Figure 3 and Table S2).

**Figure 3:**
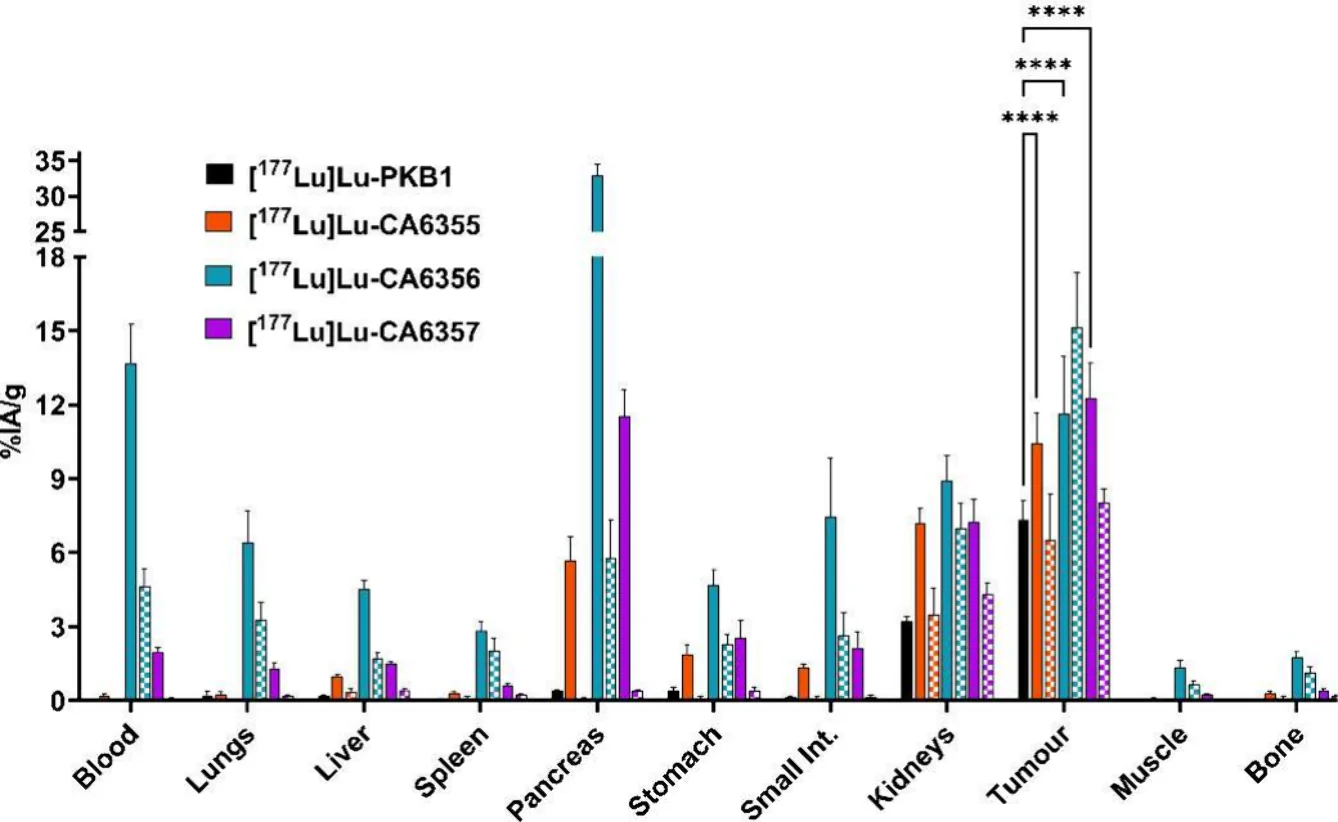
Biodistribution of [^177^Lu]­Lu-CA6355, [^177^Lu]­Lu-CA6356 and [^177^Lu]­Lu-CA6357 compared with [^177^Lu]­Lu-PKB1 in PC-3 xenografted mice. Full pattern: 4 h pi; squared pattern: 24 h pi. Data are presented as mean ± SD (*n* = 4). Statistical differences of the activity uptake in tumors at 4 h pi are indicated as **** (*p* < 0.0001).

All ABM-containing radioconjugates demonstrated significantly higher tumor uptake than the comparator [^177^Lu]­Lu-PKB1, similar to other reported GRPR-targeting conjugates incorporating ABMs.^11,15,16^ Tumor uptake 4 h pi was comparable across the series, but at 24 h pi, the two 4-(*p*-tolyl)­butyric acid-containing conjugates ([^177^Lu]­Lu-CA6355 and [^177^Lu]­Lu-CA6357) showed ∼ 1.5-fold decrease, consistent with their faster systemic clearance. In contrast, [^177^Lu]­Lu-CA6356 (containing a 4-(p-iodophenyl)­butyric acid ABM) showed a 1.2-fold increase in tumor uptake, correlating with sustained blood levels. Together, these findings indicate that ABM incorporation enhances tumor targeting, with the iodinated ABM providing the greatest effect despite all conjugates having similar GRPR affinity.

A comparable pattern was observed for pancreatic uptake with [^177^Lu]­Lu-CA6355, [^177^Lu]­Lu-CA6356 and [^177^Lu]­Lu-CA6357 showing significantly higher pancreatic levels than [^177^Lu]­Lu-PKB1. Washout was rapid for [^177^Lu]­Lu-CA6355 and [^177^Lu]­Lu-CA6357, whereas [^177^Lu]­Lu-CA6356 maintained pancreatic retention, consistent with sustained systemic circulation. ABM-containing constructs also resulted in higher kidney uptake compared to the control peptide. Importantly, however, tumor uptake exceeded kidney levels at both 4 and 24 h pi for all new analogues. This elevated renal activity is consistent with previous reports on ABM-bearing GRPR-targeting conjugates, including [^99m^Tc]­[Tc­(CO)_3_L^24^] and [^111^In]­In-AMTG-DA1 ^15,16^ and likely reflects slower renal elimination relative to parent peptides.

Across other healthy tissues, all radioconjugates demonstrated background activity proportional to blood levels. This pattern is consistent with the increased systemic circulation induced by ABM incorporation and aligns with previous reports on other ABM-containing molecules.^13,14,17,18,24^

The GRPR specificity of [^177^Lu]­Lu-CA6356 was also assessed in PC-3 xenografts. At 4 h pi, tumor uptake was significantly reduced by 2.4-fold following coinjection with an excess of NOTA-PEG_2_-RM26,^25^ confirming in vivo GRPR-mediated uptake (Table S2).

Given the prolonged blood retention and increased tumor accumulation of [^177^Lu]­Lu-CA6356, its biodistribution was further evaluated at 72 and 168 h pi to assess tumor retention and its therapeutic potential (Figure 4 and Table S2).

**Figure 4:**
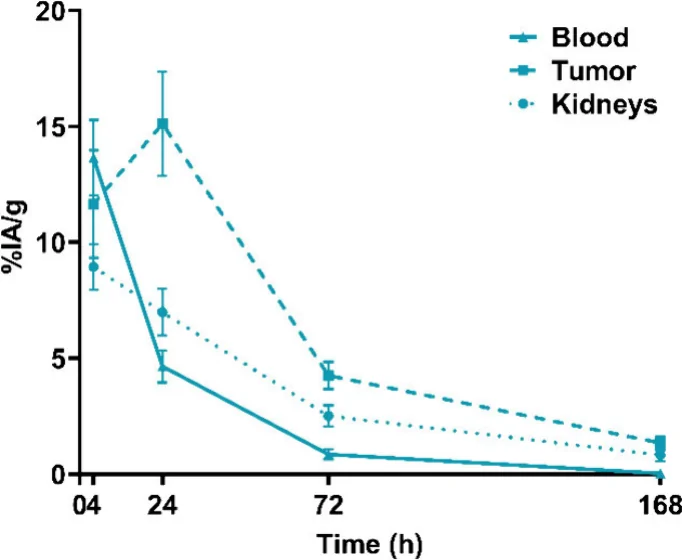
Clearance of [^177^Lu]­Lu-CA6356 from blood, tumor and kidneys over 168 h pi in PC-3 xenografted mice.

Blood activity for [^177^Lu]­Lu-CA6356 decreased over time (T_1/2_ 1.7 h), yet remained substantially longer than for other non-ABM peptides. For comparison, the circulation half-time for [^177^Lu]­Lu-DOTAGA-PEG_2_-RM26 was 0.1 h (calculated from [Mitran et al. 2019][Bibr ref26]). The circulation was extended by 2-fold in comparison with a similar peptide coupled to ABD, [^111^In]­In-DOTA-ABD-RM26 (calculated from [Abouzayed et al. 2020][Bibr ref11]).

Renal uptake for [^177^Lu]­Lu-CA6356 was higher than for [^177^Lu]­Lu-PKB1, but remained moderate compared to [^111^In]­In-DOTA-ABD-RM26 and lower than tumor uptake at all time points. Consistent with this, kidney clearance was much slower (T_1/2_ 45 h) than for [^177^Lu]­Lu-DOTAGA-PEG_2_-RM26 (T_1/2_ 0.16 h, calculated from [Mitran et al. 2019][Bibr ref26] ).

The tumor activity uptake reached a maximum at 24 h pi and thereafter decreased with time (T_1/2_ 20.5 h). This contrasts with the prolonged tumor retention observed for [^111^In]­In-DOTA-ABD-RM26, but is somewhat similar to the ABM-containing construct, [^111^In]­In-AMTG-DA1.^11,16^ Notably, the uptake profile of [^111^In]­In-DOTA-ABD-RM26, characterized by continuous accumulation over time, resembles that observed for [^177^Lu]­Lu-CA6353 and [^177^Lu]­Lu-CA6355 despite their lower GRPR affinity (IC_50_ 30–50 nM). Between 24 and 72 h pi, kidney activity for [^177^Lu]­Lu-CA6356 decreased 2.3-fold, whereas tumor uptake decreased 3.8-fold, indicating that prolonged circulation does not directly translate into sustained tumor retention.

The faster washout of activity from tumors relative to the kidneys resulted in a tumor-to-kidney ratio of 1.65 (calculated based on area under the curve). This is lower than reported values for [^177^Lu]­Lu-DOTAGA-PEG_2_-RM26 (∼2) and [^177^Lu]­Lu-AU-SAR-M1 (5.4)^26,27^ and is comparable to the ratio observed for [^111^In]­In-AMTG-DA1 (1.7).^16^

Comparison of [^177^Lu]­Lu-CA6356 with previously reported albumin-binding constructs ([^111^In]­In-DOTA-ABD-RM26 and [^111^In]­In-AMTG-DA1) as well as our recently reported non-ABM analogues ([^177^Lu]­Lu-PKB2 and [^177^Lu]­Lu-PKB3) highlights the multifactorial nature of GRPR radioligand performance. Although [^177^Lu]­Lu-CA6356 exhibited markedly prolonged blood circulation and substantially higher GRPR affinity than the non albumin-binding comparators, its tumor uptake was of a similar magnitude. Likewise, despite enhanced systemic exposure, tumor accumulation was not substantially improved relative to PKB2 and PKB3. Together, these observations indicate that neither prolonged circulation nor receptor affinity alone is sufficient to predict tumor targeting performance. Rather, tumor retention appears to be a critical determinant of overall efficacy, reflecting the complex interplay between affinity, pharmacokinetics, lipophilicity and off-target interactions. These findings emphasize the importance of balanced optimization of these parameters when developing next-generation GRPR-targeting radiotherapeutics. Future improvements will likely require iterative optimization of the peptide, linker, chelator and albumin-binding components of the construct.

In conclusion, we designed, synthesized and evaluated four novel GRPR-targeting peptides conjugated with ABMs to explore their therapeutic potential in targeted radionuclide therapy. The introduction of the ABMs preserved GRPR affinity while extending the circulation time of [^177^Lu]­Lu-labeled conjugates, with pharmacokinetics governed both by the choice of ABM as well as its placement within the construct. Notably, constructs bearing the ABM on the lysine side-chain exhibited substantially higher blood activity and retention than their N-terminal counterparts, demonstrating that ABM placement alone can strongly influence in vivo behavior. These findings demonstrate that ABM positioning, lipophilicity and size collectively modulate pharmacokinetics and tissue distribution. Overall, this work establishes ABM incorporation as a modular and tunable strategy to control systemic exposure and tissue distribution in peptide-based radiopharmaceuticals, providing a practical framework for the rational design of next-generation GRPR-targeting therapeutics.

## Supplementary Material



## Notes

Experimental section, giving synthesis and characterization data for peptide precursors, radiolabeling, in vitro and in vivo characterization methods and data, and supplementary figures and tables (PDF)

## Abbreviations

ABM: albumin-binding moiety;
ABD: albumin-binding domain;
BBN: bombesin;
DCM: dichloromethane;
DIC: *N*,*N*′-diisopropylcarbodiimide;
DIPEA: *N*,*N*-diisopropylethylamine
DMF: dimethylformamide;
DOTA: 1,4,7,10-tetraazacyclododecane-1,4,7,10-tetraacetic acid;
DOTAGA: 1,4,7,10-tetraazacyclododecane-1-glutaric acid-4,7,10-triacetic acid;
Fmoc: 9-fluorenylmethyloxycarbonyl;
Gly: glycine;
GI: gastrointestinal tract;
GRPR: gastrin-releasing peptide receptor;
HPLC: high-performance liquid chromatography;
HSA: human serum albumin;
iTLC: instant thin-layer chromatography;
KD: equilibrium dissociation constant;
NEP: neprilysin;
NMRI: Naval Medical Research Institute;
NOTA: 1,4,7-triazacyclononane-1,4,7-triacetic acid;
PC-3: Prostate Cancer Cell Line-3;
PEG: polyethylene glycol;
PET: positron emission tomography;
pi: postinjection;
PSMA: prostate-specific membrane antigen;
RP-HPLC: reversed-phase high-performance liquid chromatography;
Sar: sarcosine (*N*-methyl glycine);
SPPS: solid-phase peptide synthesis;
*t*-Bu: *tert*-butyl;
TFA: trifluoroacetic acid;
TIPS: triisopropylsilane;
TRT: targeted radionuclide therapy;
RCP: radiochemical purity.
