# Supplementary material for: Albumin-Binding Moieties Tune the Pharmacokinetics and Tumor Uptake of 177Lu-Labeled GRPR Radioligands
Source: ACS Med Chem Lett. 2026 Sep 4;17(10):2211–8. doi: 10.1021/acsmedchemlett.6c00338 (PMC13656281; doi:10.1021/acsmedchemlett.6c00338)
Supplement: Supplementary file 1 [file ml6c00338_si_001.pdf]

**SUPPORTING INFORMATION**

# Albumin-Binding Moieties Tune the Pharmacokinetics and Tumour Uptake of $^{177}$Lu-Labelled GRPR Radioligands

Karim Obeid[1‡], Esther Olaniran Håkansson[1‡], Ekaterina Bezverkhniaia[1], Bobo Skillinghaug[1], Ulrika Rosenström[1], Panagiotis Kanellopoulos[1], Anna Orlova[1,2], Luke R. Odell[1*]

[1]Department of Medicinal Chemistry, BMC Uppsala University, P.O. Box 574, SE-751 23 Uppsala, Sweden

[2]Science for Life Laboratory, Uppsala University, 752 37 Uppsala, Sweden

* luke,odell@ilk.uu.se

## Table of contents

General ........ 2
Peptide Synthesis ........ 2
Coupling and deprotection ........ 2
General procedure 1 ........ 2
General procedure 2 ........ 2
Cleavage and global deprotection ........ 2
Synthesis of CA6353 ........ 2
Synthesis of CA6355 ........ 2
Synthesis of CA6356 ........ 2
Synthesis of CA6357 ........ 2
Radiolabelling ........ 3
LogD determination ........ 3
In vitro studies ........ 3
Binding specificity ........ 3
Cellular internalization ........ 3
Affinity measurements ........ 3
Cellular retention ........ 3
In vivo studies ........ 4
In vivo stability ........ 4
Biodistribution in healthy mice ........ 4
Biodistribution in PC-3 tumour-bearing mice ........ 4
Compound characterization ........ 5
CA6353 ........ 5
CA6355 ........ 7
CA6356 ........ 9
CA6357 ........ 11
Supplementary tables ........ 16
References ........ 18

## Materials and Methods

### General

Analytical high-performance liquid chromatography (HPLC) with electrospray ionization mass spectrometry (ESI-MS) was performed using a C18 column (50 × 3.0 mm, 2.6 µm particle size, 100 Å pore size). The mobile phase consisted of $CH_3CN$ and 0.05% aqueous formic acid (HCOOH), with a flow rate of 1.5 mL/min. Preparative reversed-phase HPLC (RP-HPLC) was conducted with UV detection at 214 nm using a Glison system equipped with a Macherey-Nagel NUCLEODUR C18 HTec column (21 × 125 mm, 5 µm particle size). Elution was carried out using $H_2O/CH_3CN$ containing 0.1% trifluoroacetic acid (TFA) at a flow rate of 25 mL/min. High-resolution mass spectra (HRMS) were acquired using a matrix-assisted laser desorption/ionization (MALDI) Solarix FTICR instrument (Bruker Daltonics, Bremen, Germany) operating in positive ion mode. 2,5-Dihydroxybenzoic acid (DHB) was used as the matrix at a concentration of 35 mg/mL, prepared in 50% acetonitrile containing 0.1% trifluoroacetic acid (TFA). All chemicals and solvents were purchased from Sigma-Aldrich, Fisher Scientific, or VWR and used without further purification. PKB1 and NOTA-$PEG_2$-RM26 were purchased from Pepmic Co., Ltd. (Suzhou, China). Lu-177 was obtained as [$^{177}$Lu]$LuCl_3$ from ITM Isotope Technologies (Munich, Germany). PC-3 (GRPR+) human cell line (2.5-3.5 × $10^5$ receptors/cell[1]) was provided by American Type Culture Collection (Manassas, VA, USA). They were maintained with Roswell Park Memorial Institute (RPMI) 1640 medium (+ 10% fetal bovine serum and +1% penicillin 100 IU/mL and streptomycin 100 µg/mL). Trypsin-EDTA 0.25% (Biochrom AG, Berlin, Germany) was used for cellular detachment. The radioactivity content of the collected samples was measured with the Wizard2TM gamma counter (PerkinElmer, Waltham, MA, USA).

### Peptide Synthesis

Peptide conjugates were synthesized by manual solid-phase peptide synthesis (SPPS) as illustrated in **Schemes 1**. The synthesis was performed on Fmoc Rink Amide 4-Methylbenzhydrylamine (MBHA) resin (loading 0.76 mmol/g) on a 0.1 mmol scale, using 12 mL disposable syringes fitted with filters. The resin was pre-swollen in DCM for 40 minutes. Fmoc deprotection was performed with 20% piperidine in DMF (3 × 10 min) followed by washing with DMF (6 × 1 min). Standard side-chain protecting groups were used: Trt for His and Gln, and Boc for Trp.

**Coupling and deprotection.** Fmoc-protected amino acids (4 equiv.) were coupled using Oxyma (4 equiv.), DIC (4 equiv.), and DIPEA (7 equiv.) in DMF (2 mL) for 2 h. For coupling Fmoc-Sta-OH (2 equiv.), the equivalents of reagents were halved and the reaction was allowed to proceed overnight. Fmoc-4-amino-1-carboxymethyl-Piperidine was coupled in 4 mL DMF to improve solubility and the reaction was left overnight. After each coupling step, the resin was washed with DMF (6 × 1 min), and Fmoc removal was repeated using 20% piperidine in DMF (3 × 10 min), followed by additional washes with DMF (6 × 1 min). Once the desired sequence 1 was assembled, general procedures 1 or 2 were employed and final cleavage and deprotection yielded conjugates CA6353, CA6355 and CA6357.

**General procedure 1**: Following Fmoc deprotection of *N*-protected lysine, ABM 1 (4-(*p*-iodophenyl)butanoic acid) or ABM 2 (4-(*p*-tolyl)butanoic acid) was coupled (4 equiv.) according to **Scheme 1, Route A**. After assembly of the full sequence, the Alloc protecting group was removed using $Pd(PPh_3)_4$ (0.3 equiv.) and phenylsilane ($PhSiH_3$, 20 equiv.) in DCM (2 mL). Notably, this step led to complete deiodination in the construct containing ABM 1 (see Section 3). The liberated amino group on lysine was then conjugated with DOTA-tris(*t*Bu-ester) (4 equiv.) under standard coupling conditions.

**General procedure 2**: In this alternative route, Alloc deprotection of lysine was first performed using $Pd(PPh_3)_4$ (0.3 equiv.) and $PhSiH_3$ (20 equiv.) in DCM (2 mL). The appropriate ABM (4 equiv.) was then introduced, as outlined in **Scheme 1, Route B**. Subsequently, the *N*-terminus was deprotected and DOTA-tris(*t*Bu-ester) (4 equiv.) was coupled under standard conditions.

**Cleavage and global deprotection.** Peptides were cleaved from the resin and globally deprotected using a TFA-based cocktail (TFA:TIS:$H_2O$, 95:2.5:2.5) for 2-3 hours at room temperature. Crude peptides were precipitated by the addition of cold diethyl ether, centrifuged and washed twice with cold diethyl ether. Purification was performed by preparative RP-HPLC using 0.1% TFA in $H_2O$ and 0.1% TFA in MeCN as eluents. Lyophilization of purified fractions yielded the final peptides as colourless solids. Purity was confirmed by analytical RP-HPLC-MS and identity was verified by HRMS.

**Synthesis of CA6353.** General procedure 1 was followed using ABM 1 (4-(*p*-iodophenyl)butanoic acid) (4 equiv.). Deiodination yielded title conjugate CA6353 (**Scheme 1, Route A** and **Figure 1**) in 7% yield (16 mg). Purity was confirmed by RP-HPLC-MS (**Figure S1 in Supporting Information)**. Identity was confirmed by HRMS (MALDI) m/z $[M + H]^+$ calculated for $C_{101}H_{154}N_{23}O_{24}$ 2074.15; found 2074.16282 (**Figure S2**).

**Synthesis of CA6355.** General procedure 1 was followed using ABM 2 (4-(*p*-tolyl)butanoic acid) (4 equiv.), yielding title conjugate CA6355 (**Scheme 2, Route A** and **Figure 1**) in 9% yield (20 mg). Purity was confirmed by RP-HPLC-MS (**Figure S3)**. Identity was confirmed by HRMS (MALDI) m/z $[M + H]^+$ calculated for $C_{102}H_{156}N_{23}O_{24}$ 2088.16; found 2088.17645 (**Figure S4**).

**Synthesis of CA6356.** General procedure 2 was followed using ABM 1 (4-(*p*-iodophenyl)butanoic acid) (4 equiv.), yielding title conjugate CA6356 (**Scheme 2, Route B** and **Figure 1**) in 8% yield (18 mg). Purity was confirmed by RP-HPLC-MS (**Figure S5)**. Identity was confirmed by HRMS (MALDI) m/z $[M + H]^+$ calculated for $C_{101}H_{153}IN_{23}O_{24}$ 2200.05; found 2200.05351 (**Figure S6**).

**Synthesis of CA6357.** General procedure 2 was followed using ABM 2 (4-(*p*-tolyl)butanoic acid) (4 equiv.), yielding title conjugate CA6357 (**Scheme 1, Route B** and **Figure 1**) in 7% yield (16 mg). Purity was confirmed by RP-HPLC-MS (**Figure S7)**. Identity was confirmed by HRMS (MALDI) m/z $[M + H]^+$ calculated for $C_{102}H_{156}N_{23}O_{24}$ 2088.16; found 2088.18161 (**Figure S8**).

## Radiolabelling

Radiolabelling of CA6353 (n = 8), CA6355 (n = 9), CA6356 (n = 9), CA6357 (n = 9) and PKB1 (n = 2) was performed by incubating 5 - 125 MBq [$^{177}$Lu]LuCl$_3$ with 2-5 nmol of peptide, 20 µL EtOH (99.5%), and 40 µL $NH_4$Ac buffer (0.2 M, pH 5.5). The mixture was incubated at 85°C for 30 minutes. The radiochemical purity was analysed on RP-HPLC equipped with a flow-scan radioactivity detector (Bioscan) with an FC-3300 NaI/PMT radioactivity probe (Eckert & Ziegler, Berlin, Germany), LaPrep Sigma HPLC LP1100 pump (Hitachi High-Tech Corporation, Hitachinaka, Ibaraki, Japan), a manual Rheodyne 7725i injector fitted with a 20 µL loop (IDEX Health & Science, LLC, Rohnert Park, CA, USA), a 40D LWL UV detector with a 4 µL flow cell (Knauer, Berlin, Germany). A Luna C18 column was used (5 µm, 100 Å, 150 × 4.6 mm, Phenomenex, Værløse, Denmark). The elution gradient started at 95% 0.1% v/v aqueous trifluoroacetic acid (TFA, solvent A) / 5% 0.1% v/v TFA in acetonitrile (MeCN, solvent B), and over 20 minutes it reached 40% TFA/60% MeCN. Radiochemical yields were determined by instant thin-layer chromatography (iTLC) using silica gel-impregnated glass microfiber strips (Agilent Technologies, Santa Clara, CA, USA) as the stationary phase and 0.2 M citric acid as the mobile phase (n = 8 for [$^{177}$Lu]Lu-CA6353, n = 9 for [$^{177}$Lu]Lu-CA6355, [$^{177}$Lu]Lu-CA6356 and [$^{177}$Lu]Lu-CA6357, and n = 2 for [$^{177}$Lu]Lu-PKB1). The yields were determined using Cyclone® Plus Phosphorimager (PerkinElmer, Hägersten, Sweden).

## LogD determination

To determine the octanol-water distribution coefficient (LogD), 10 pmol of radiopeptide was added to an Eppendorf tube containing 500 µL n-octanol and 500 µL Milli-Q water. The tube was vortexed for 2 min, and then centrifuged at high speed for 5 min. Three 100 µL aliquots were sampled from both water and octanol phases. Radioactivity in each aliquot was measured using the Wizard2TM gamma counter (Revvity, Hägersten, Sweden) and LogD was calculated.

## In vitro studies

**Binding specificity.** To evaluate the GRPR specificity of the radiolabelled peptides, 1 × $10^6$ PC-3 cells/well were seeded in 6-well plates (VWR International, Radnor, USA). The next day, the culture medium was discarded, and the cells were washed with cold (4°C) phosphate-buffered saline containing 1% w/v bovine serum albumin (PBS/BSA). The radiopeptide under evaluation (diluted in PBS/BSA) was added to all wells at a final concentration of 1 nM. A subset of wells was also treated with a 1000-fold molar excess of unlabelled NOTA-PEG2-RM26[2] to block GRPR. Following a 1-hour incubation at 37°C, the supernatant was removed, and the cells were rinsed and then collected by treating them twice with 500 µL 1 M NaOH. The activity of the collected samples was measured with the gamma counter. This assay was performed in triplicate for each radioconjugate. Statistical analysis was performed using two-way ANOVA with Tukey's post hoc test in GraphPad Prism 10 (GraphPad Software, Boston, MA, USA).

**Cellular internalization.** To assess the cellular internalization of the radioconjugates, 1 × $10^6$ PC-3 cells were seeded in 35 mm Petri dishes (Corning Incorporated, New York, USA). After the medium was removed and the cells were washed, the test radioconjugate was added to each dish ($C_f$ = 1 nM), and the dishes were incubated at 37°C. At 1h, 4 h, and 24 h time points, the cells were washed with cold PBS/BSA and incubated for 5 minutes on ice with an acidic solution (0.2 M glycine buffer with 0.15 M NaCl and 4 M urea, pH 2) to collect the supernatant (membrane-bound fraction). After repeating this last step, the cells were washed with PBS/BSA and treated twice with 0.1 M NaOH and collected (internalized fraction). The activity content of the collected samples was quantified using the gamma counter. Three repetitions per time point were performed.

**Affinity measurements.** The GRPR affinity of each radiopeptide was determined in the LigandTracer instrument (Ridgeview Instruments AB, Uppsala, Sweden). 3 × $10^6$ PC-3 cells were seeded on one side of 100 mm Petri dishes (Corning Incorporated, New York, USA). The radioconjugates were tested at 1 nM and 3 nM concentrations to get the association curves. After reaching the plateau, the media containing radiopeptides was replaced with fresh complete media to measure the dissociation curve overnight. The sensorgrams were analysed using the TraceDrawer software (Ridgeview Instruments AB, Uppsala, Sweden), and the curves were fitted with a 1:1 model.

**Cellular retention.** One day before the experiment, 1 × $10^6$ PC-3 cells were seeded in 35 mm Petri dishes. On the day of the experiment, cells were washed with PBS and incubated with 1 mL of complete medium containing the radioconjugates under evaluation ($C_f$ = 1 nM). After 1h incubation at 37°C, the radioactive medium was aspirated, and the cells were washed with PBS. One set of wells was treated twice with 0.1 M NaOH, and cell lysates were collected. The remaining wells were incubated at 37°C with 1 mL of plain medium. At 4 h and 24 h, cells were washed with PBS/BSA, treated twice with 0.1 M NaOH, and collected. The radioactivity of all samples was measured using the gamma counter. Experiments were performed in triplicate for each conjugate and each time point.

## In vivo studies

The in vivo experiments were carried out by European standards for laboratory animal welfare under the permit number 00473/21, which is approved by the regional ethics committee for animal research (Uppsala, Sweden).

**In vivo stability.** The in vivo stability of [$^{177}$Lu]Lu-CA6353, [$^{177}$Lu]Lu-CA6355, [$^{177}$Lu]Lu-CA6356, and [$^{177}$Lu]Lu-CA6357 was assessed in healthy NMRI mice. Each radiopeptide was injected intravenously (50 MBq, 2 nmol of peptide in 100 µL PBS/BSA). Five minutes post-injection (pi), the mice were euthanized, and the blood was collected and immediately mixed with EDTA (0.1 mM, 40 µL) in Eppendorf tubes on ice. The mixture was spun at 2000 g for 10 min at 4°C in an Eppendorf Centrifuge 5430 R (Eppendorf AG, Hamburg, Germany), and the plasma was collected and diluted with an equal volume of MeCN (4°C). After a second centrifugation at 15,000 g and 4°C for 10 min, the supernatant was collected in an Eppendorf tube and concentrated to 50–100 µL at 50°C using a mild stream of N2. PBS was added to the samples to reach a final volume of 200 µL, and the solutions were filtered through a syringe filter (0.22 µm, 13 mm, VWR International, Radnor, USA). The filtered solution was analyzed by RP-HPLC to determine the percentage of intact peptides. The peak corresponding to the intact peptide was identified by injection of an aliquot of the labelling solution. Representative radiochromatograms for the four radioconjugates are given in **Figures S14-17**.

**Biodistribution in healthy mice.** The biodistribution of [$^{177}$Lu]Lu-CA6353, [$^{177}$Lu]Lu-CA6355, [$^{177}$Lu]Lu-CA6356, and [$^{177}$Lu]Lu-CA6357 was performed on healthy NMRI mice (**Table S1**). The animals were injected in the tail vein with radiopeptide diluted in PBS/BSA (100 kBq, 100 pmol, the amount of peptide was adjusted with the cold conjugate). At 1 h and 4 h pi, the animals were euthanized, and the organs (blood, liver, pancreas, kidneys, and the body) were collected and weighed, and their radioactivity content was measured in the gamma counter. Four animals were used for each time point. Results are presented as the percentage of injected activity per gram (%IA/g) for the collected organs, and percentage of injected activity (%IA) for the body. Biodistribution at 1h pi of [$^{177}$Lu]Lu-PKB1 was also performed as a comparator in the same way described above. Statistics were done using two-way ANOVA with Šidák correction using GraphPad Prism 10.

**Biodistribution in PC-3 tumour-bearing mice.** The biodistribution of [$^{177}$Lu]Lu-CA6355, [$^{177}$Lu]Lu-CA6356, and [$^{177}$Lu]Lu-CA6357 was evaluated on Balb/c nu/nu mice. The mice were subcutaneously injected on the right hind with $8 \times 10^6$ PC-3 cells. Four weeks later, the mice were injected in the tail vein with radiopeptide diluted in PBS/BSA (100 kBq, 100 pmol). At 4 h and 24 h pi, the mice were euthanized, and the organs of interest were collected, weighed, and their radioactivity content was measured in the gamma counter. Results are presented as the percentage of injected activity per gram (%IA/g) for the collected organs, and percentage of injected activity (%IA) for the gastrointestinal tract (GI) and the body. Biodistribution of [$^{177}$Lu]Lu-CA6356 was also evaluated at 72 h and 168 h pi. An additional group was included to determine the in vivo specificity of [$^{177}$Lu]Lu-CA6356 by co-injecting a 100-fold molar excess of unlabelled NOTA-PEG$_2$-RM26[1]. Biodistribution at 4 h pi of [$^{177}$Lu]Lu-PKB1 was also performed as a comparator in the same way described above. Four animals were used for each time point per peptide. Statistics were performed using two-way ANOVA with Šidák correction in GraphPad Prism 10.

# Compound characterization

## CA6353

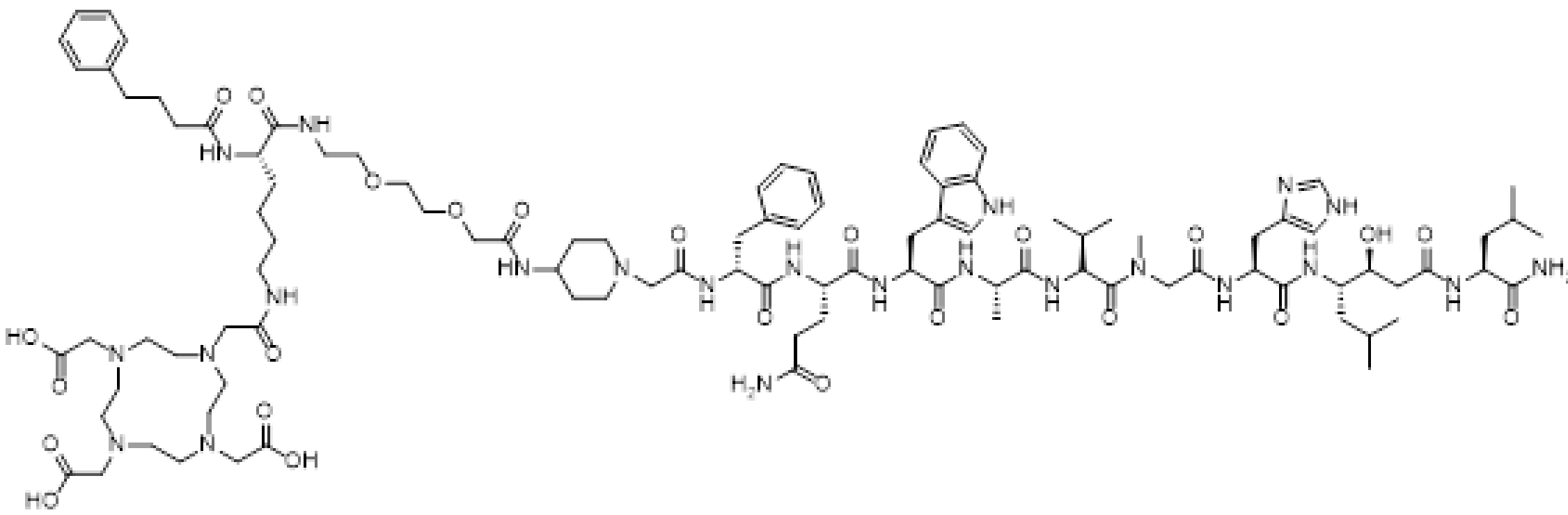

**CA6353_Pool 1**

| | | | |
|---|---|---|---|
| *Sequence Name:* | 2025_w05 | *Injection Volume:* | 10.00 |
| *Instrument Method:* | 5-100pct_positive_3min | *Vial Number:* | RA1 |
| *Processing Method:* | General_ILK | *Injection Date/Time:* | 250203 09:15 |

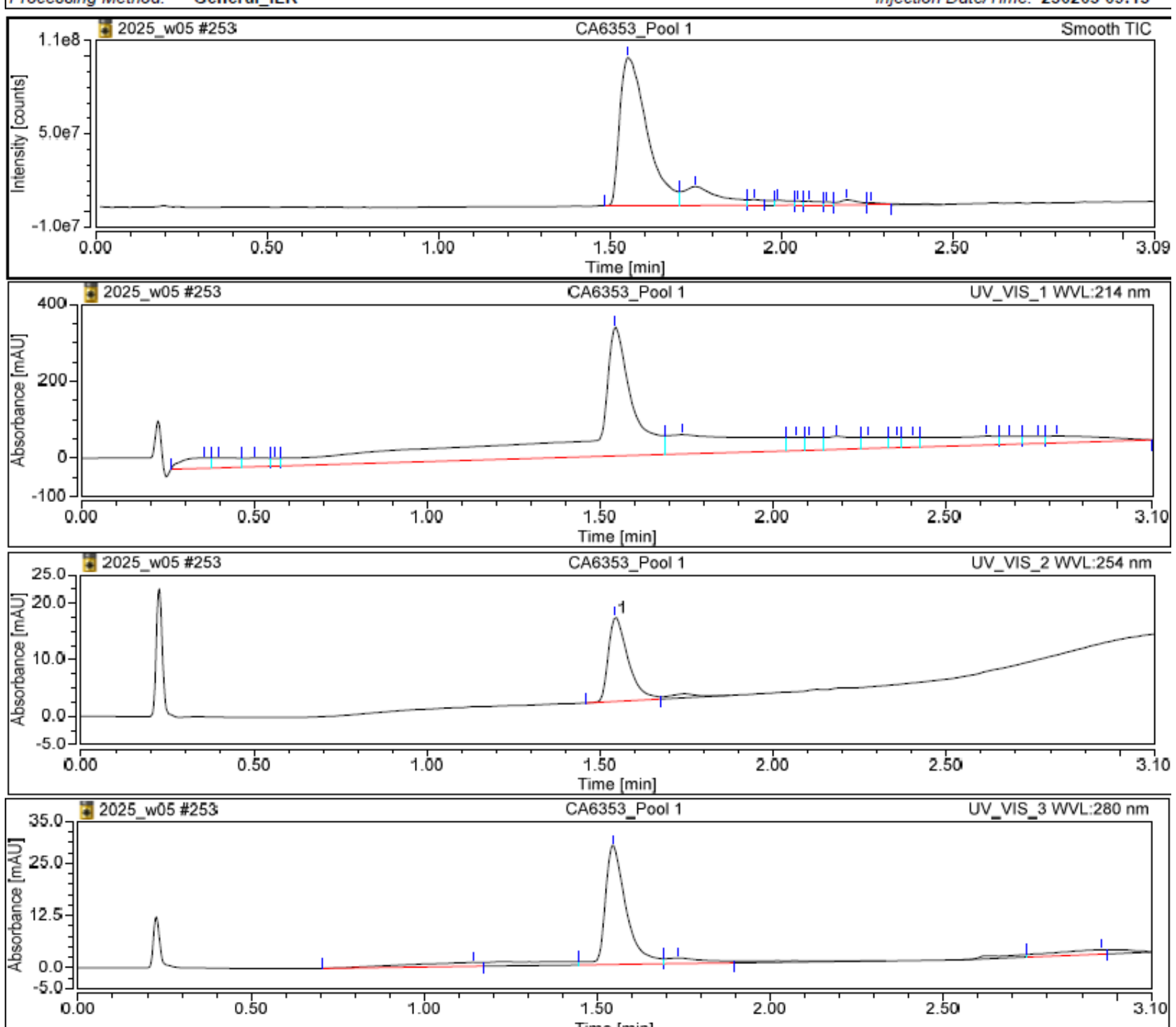


| No. | Retention Time min | Height mAU | Area mAU*min | Relative Area % | Mass 1 m/z | Mass 2 m/z | Mass 3 m/z |
|---|---|---|---|---|---|---|---|
| 1 | 1.543 | 14.907 | 0.977 | 100.00 | 692.4 | 691.6 | 1037.1 |
| Total: | | 14.907 | 0.977 | 100.00 | | | |

**Figure S1**. Analytical RP-HPLC chromatogram of CA6353 on a C18 column, using a 3-minute gradient of 5-100 % acetonitrile with 0.05% formic acid. From top to bottom: total ionization chromatogram (TIC) followed by UV detection at 214, 254 and 280 nm.

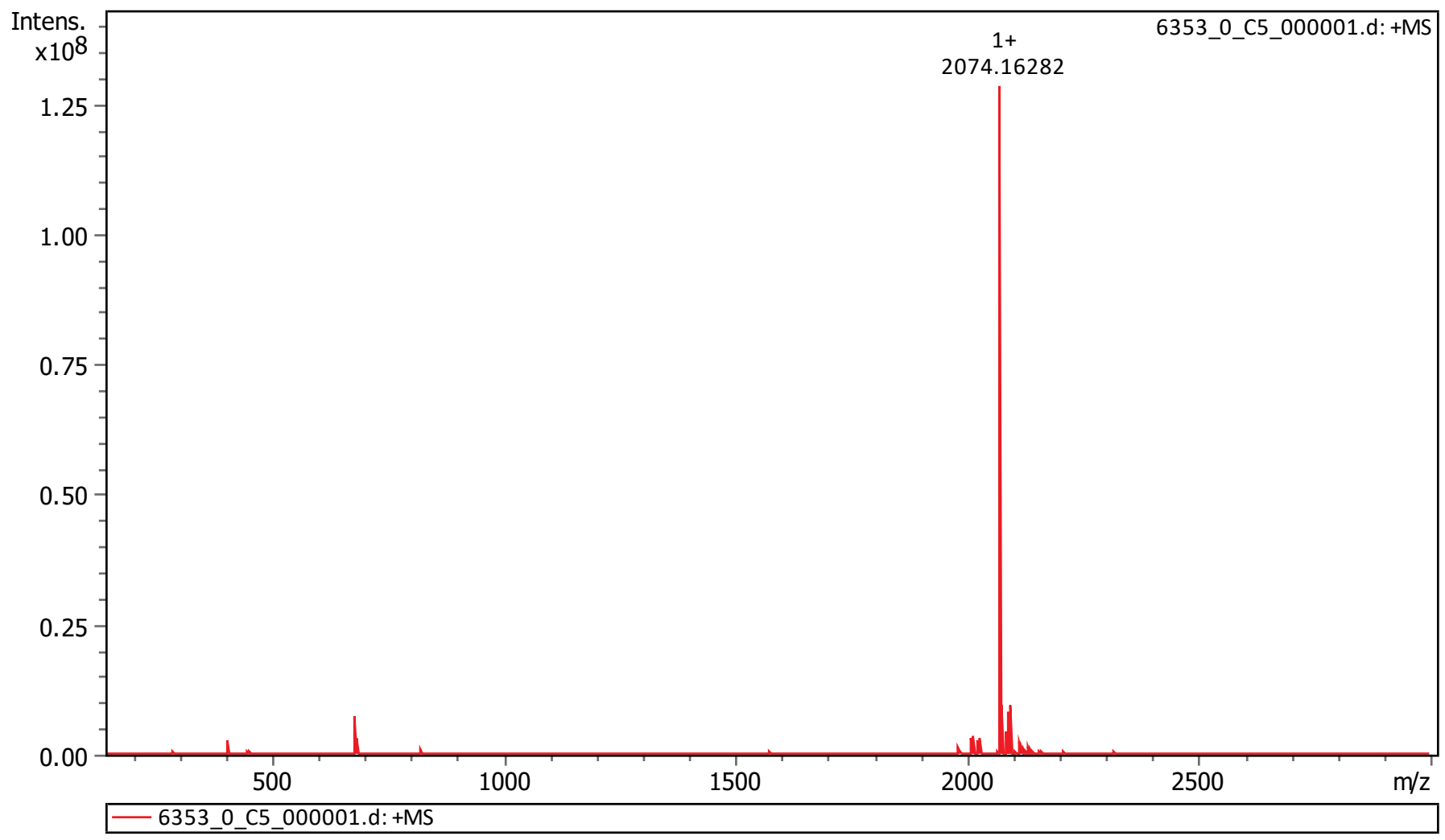


**Figure S2**. High-resolution mass spectra (HRMS) of CA6353, acquired using a matrix-assisted laser desorption/ionization (MALDI) in positive ion mode. Calculated for $C_{101}H_{154}N_{23}O_{24}$ 2074.15; found 2074.16282.

## CA6355

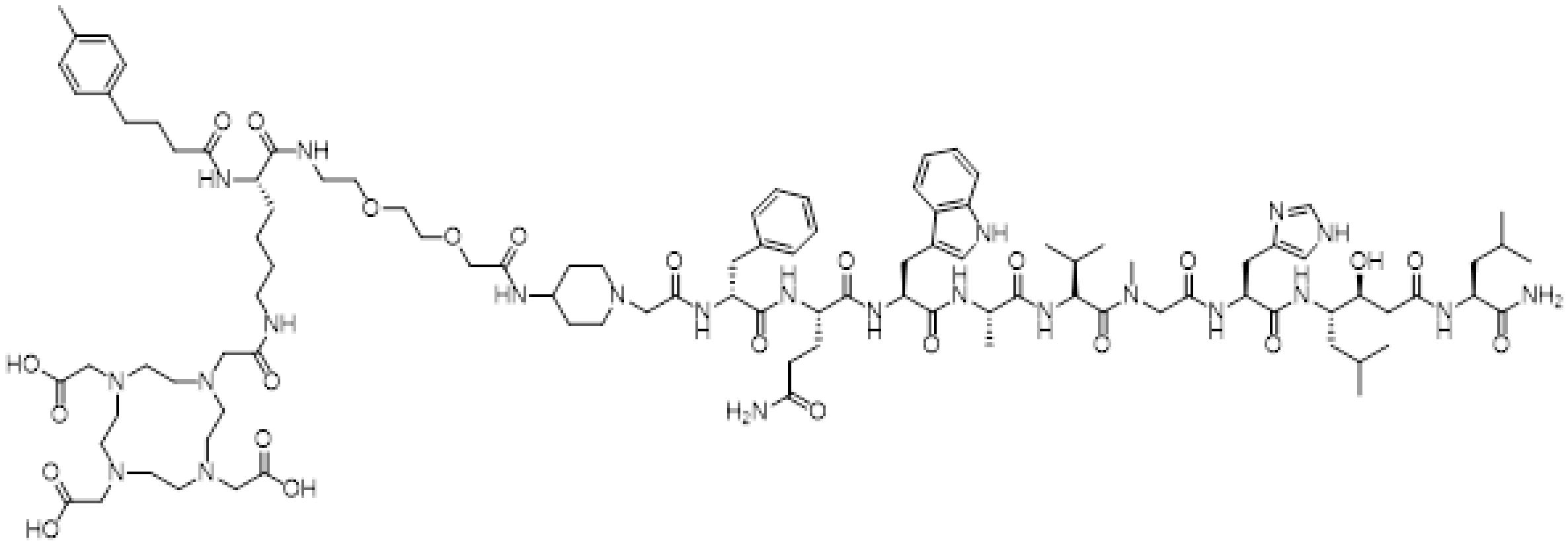

**CA6355_HPLC_pool 3**

| | | | |
|---|---|---|---|
| *Sequence Name:* | **2025_w06** | *Injection Volume:* | **10.00** |
| *Instrument Method:* | **5-100pct_positive_3min** | *Vial Number:* | **GC4** |
| *Processing Method:* | **General_ILK** | *Injection Date/Time:* | **250206 17:42** |

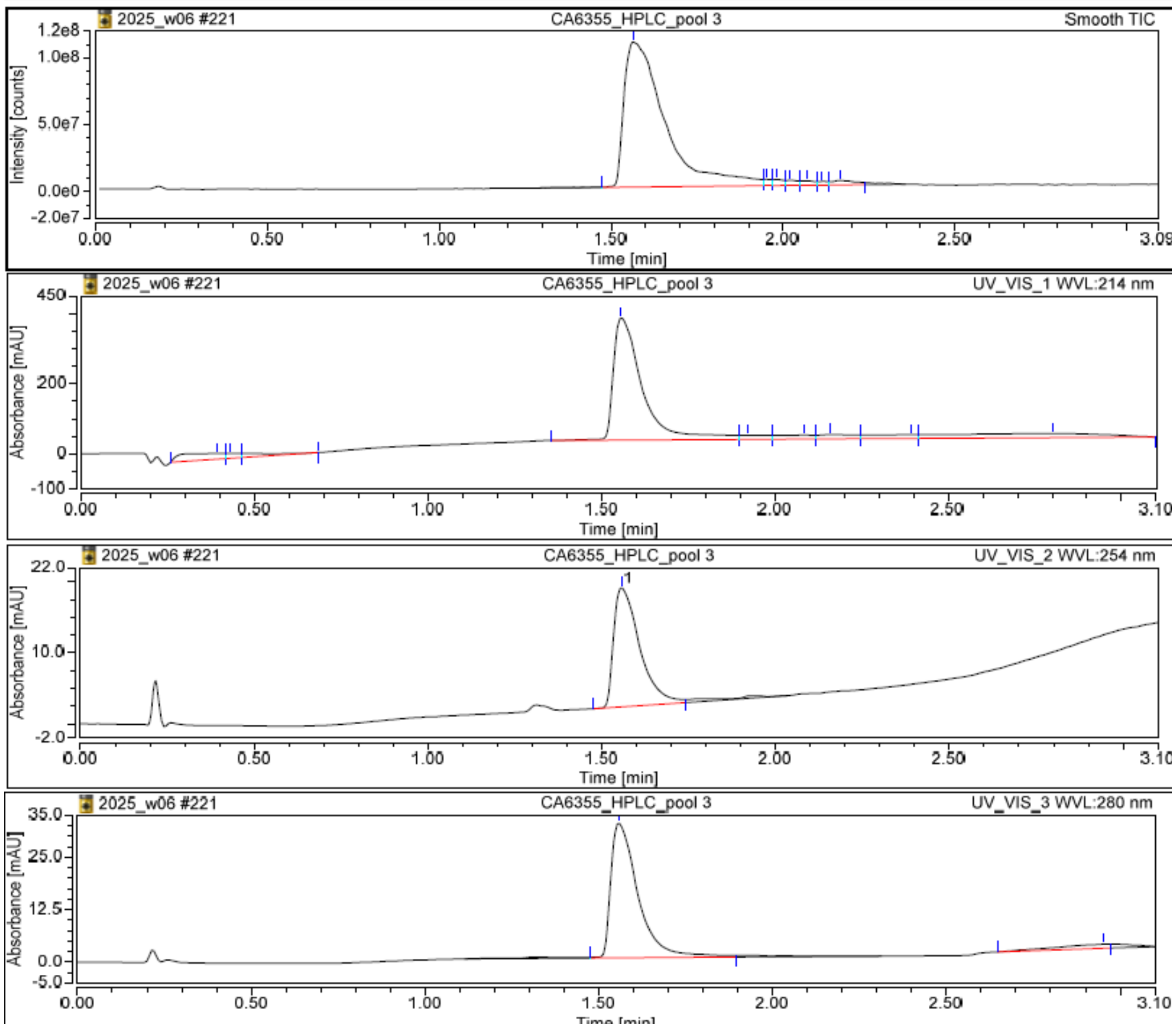


| No. | Retention Time | Height | Area | Relative Area | Mass 1 | Mass 2 | Mass 3 |
|---|---|---|---|---|---|---|---|
| | min | mAU | mAU*min | % | m/z | m/z | m/z |
| 1 | 1.557 | 16.830 | 1.445 | 100.00 | 696.8 | 1045.0 | 1043.9 |
| **Total:** | | **16.830** | **1.445** | **100.00** | | | |

**Figure S3**. Analytical RP-HPLC chromatogram of CA6355 on a C18 column, using a 3-minute gradient of 5-100% acetonitrile with 0.05% formic acid. From top to bottom: total ionization chromatogram (TIC) followed by UV detection at 214, 254 and 280 nm.

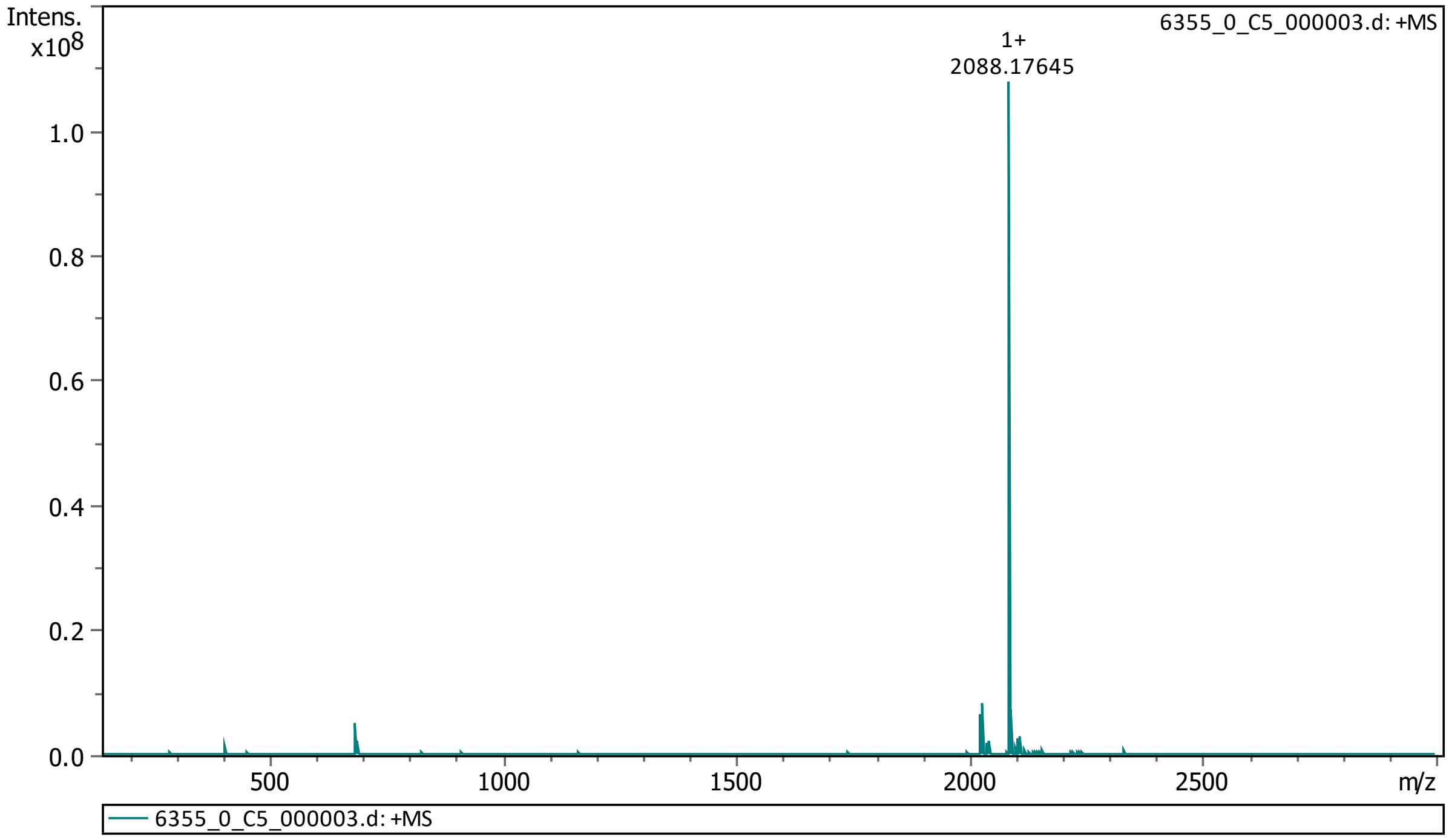


**Figure S4**. High-resolution mass spectra (HRMS) of CA6355, acquired using a matrix-assisted laser desorption/ionization (MALDI) in positive ion mode. Calculated for $C_{102}H_{156}N_{23}O_{24}$ 2088.16; found 2088.17645.

## CA6356

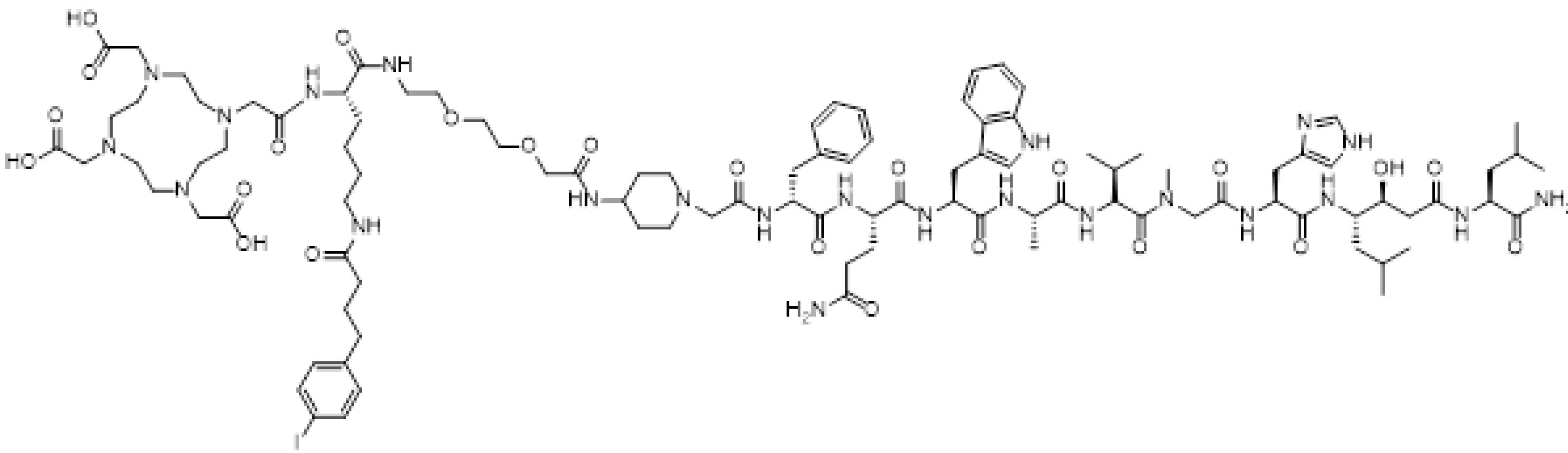


### CA6356_HPLC_pool 3

| | | | |
|---|---|---|---|
| *Sequence Name:* | 2025_w06 | *Injection Volume:* | 10.00 |
| *Instrument Method:* | 5-100pct_positive_3min | *Vial Number:* | GD4 |
| *Processing Method:* | General_ILK | *Injection Date/Time:* | 250206 17:05 |

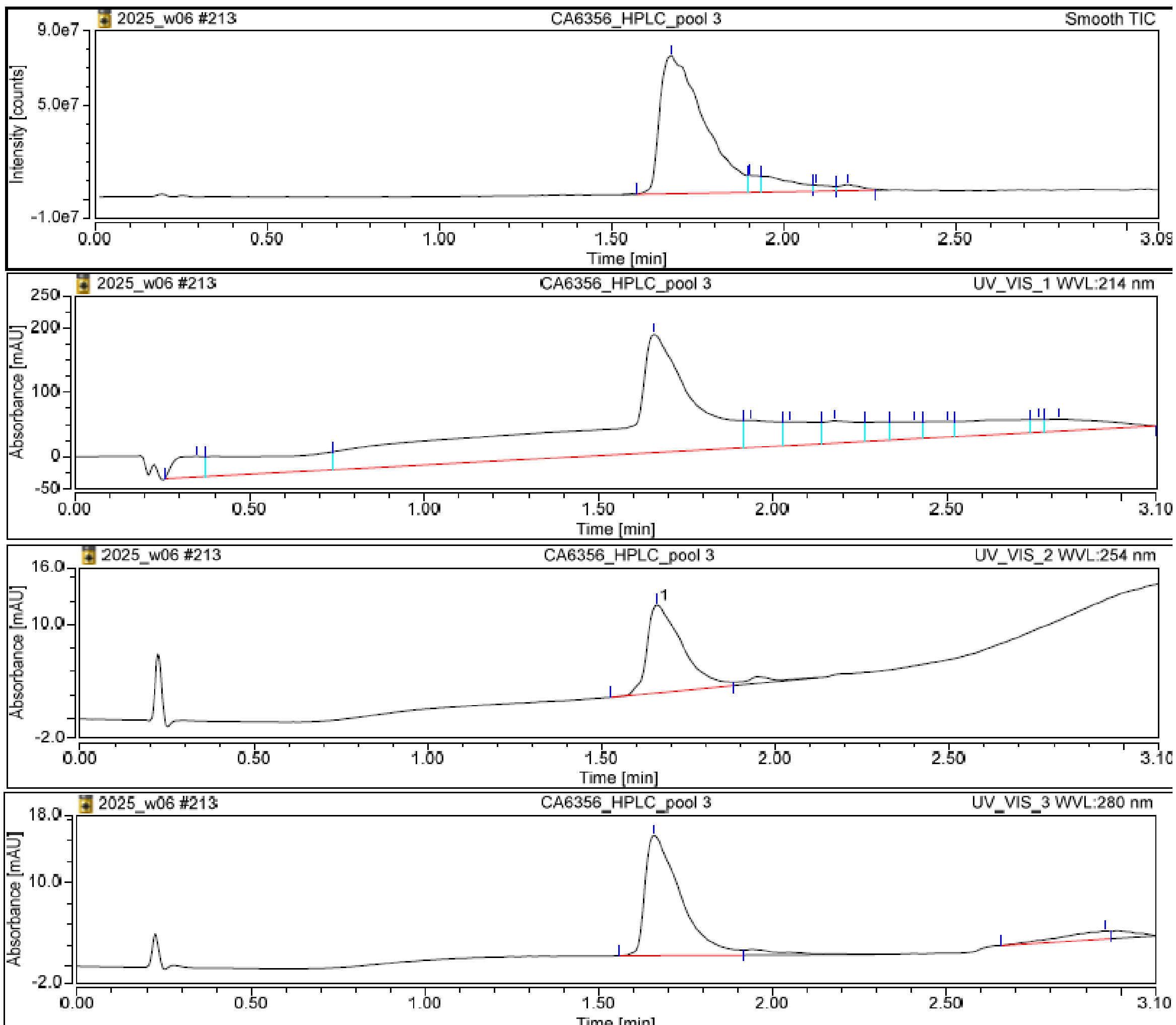


| No. | Retention Time min | Height mAU | Area mAU*min | Relative Area % | Mass 1 m/z | Mass 2 m/z | Mass 3 m/z |
|---|---|---|---|---|---|---|---|
| 1 | 1.660 | 9.297 | 1.088 | 100.00 | 734.1 | 1100.3 | 1101.4 |
| **Total:** | | **9.297** | **1.088** | **100.00** | | | |

**Figure S5**. Analytical RP-HPLC chromatogram of CA6356 on a C18 column, using a 3-minute gradient of 5-100% acetonitrile with 0.05% formic acid. From top to bottom: total ionization chromatogram (TIC) followed by UV detection at 214, 254 and 280 nm

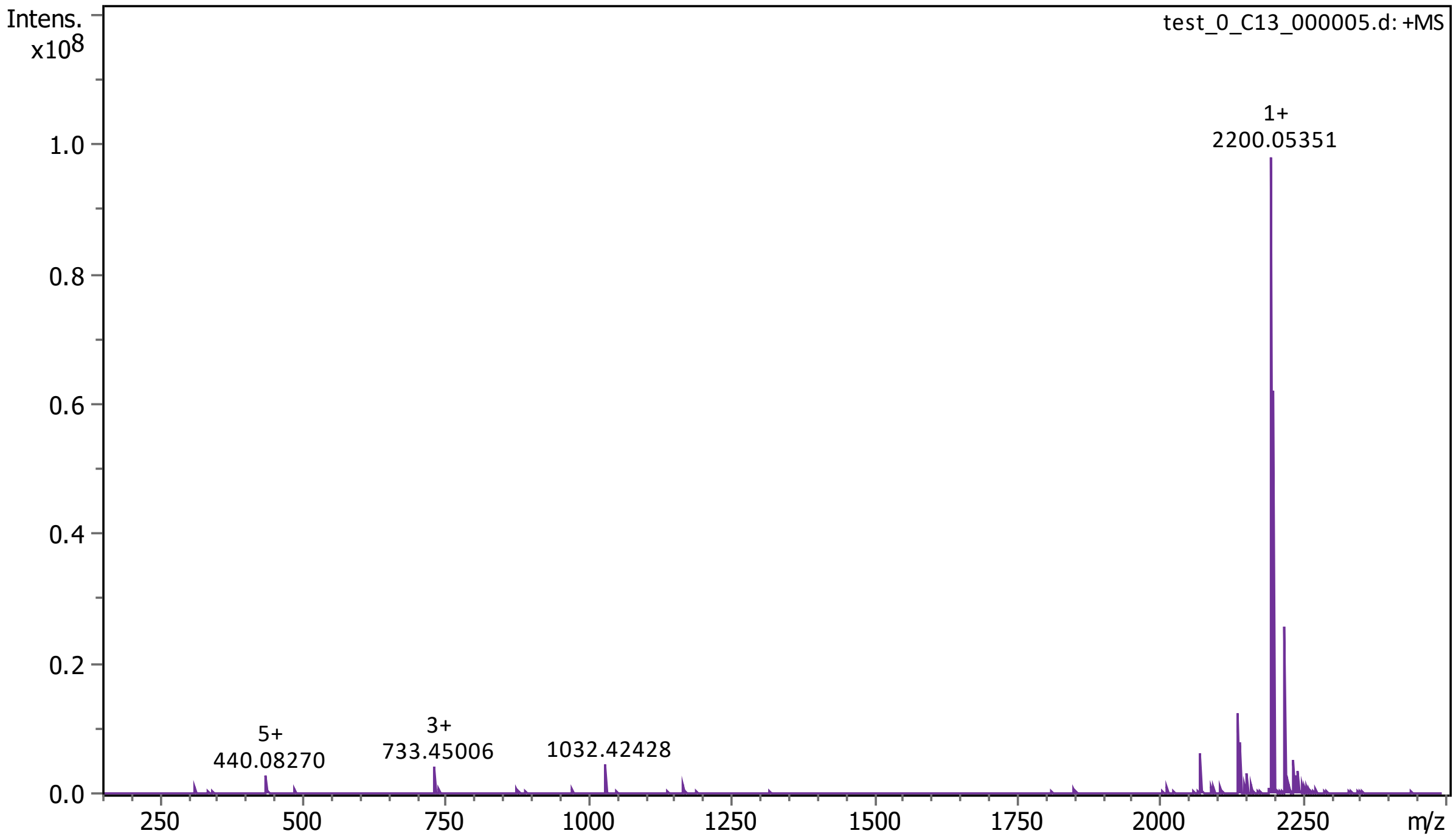


**Figure S6**. High-resolution mass spectra (HRMS) of CA6356, acquired using a matrix-assisted laser desorption/ionization (MALDI) in positive ion mode. Calculated for $C_{101}H_{153}IN_{23}O_{24}$ 2200.05; found 2200.07202.

## CA6357

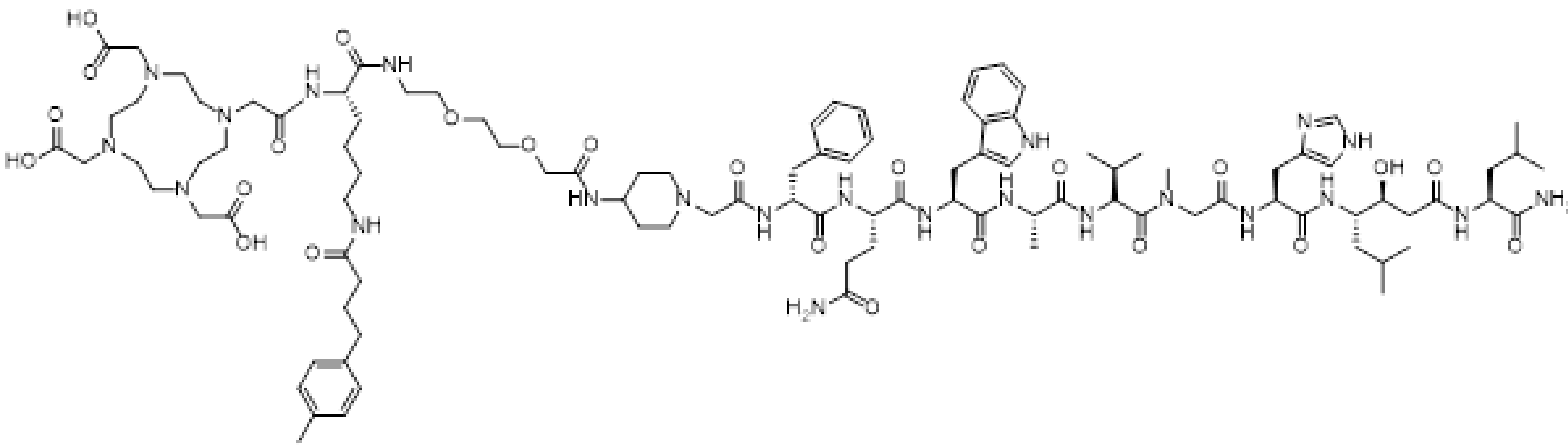


**CA6357_HPLC_pool 1**

| | | | |
|---|---|---|---|
| *Sequence Name:* | **2025_w05** | *Injection Volume:* | **5.00** |
| *Instrument Method:* | **5-100pct_positive_3min** | *Vial Number:* | **GA6** |
| *Processing Method:* | **General_ILK** | *Injection Date/Time:* | **250128 17:23** |

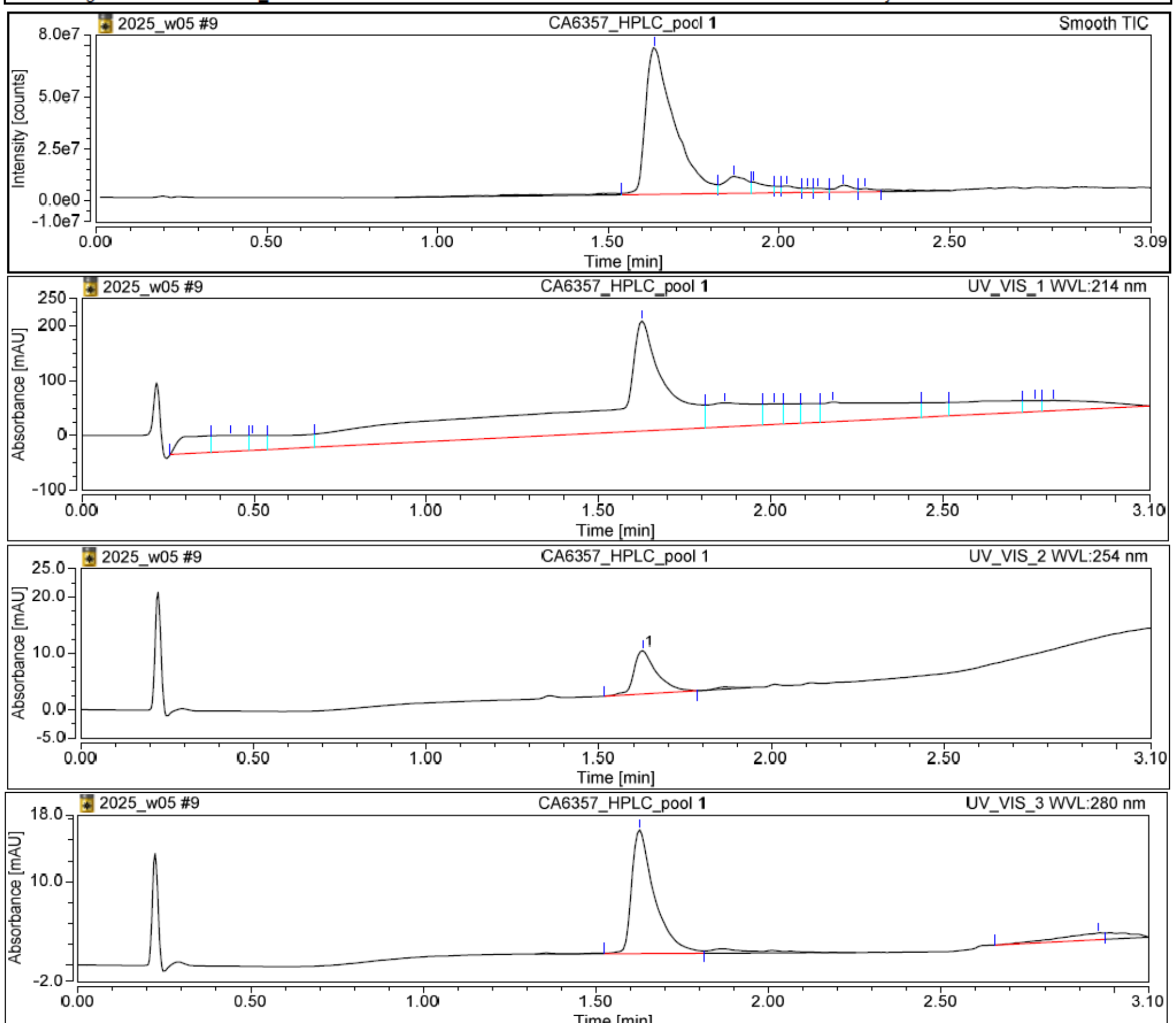


| No. | Retention Time min | Height mAU | Area mAU*min | Relative Area % | Mass 1 m/z | Mass 2 m/z | Mass 3 m/z |
|---|---|---|---|---|---|---|---|
| 1 | 1.627 | 7.667 | 0.581 | 100.00 | 696.8 | 1044.7 | 1046.2 |
| **Total:** | | **7.667** | **0.581** | **100.00** | | | |

**Figure S7**. Analytical RP-HPLC chromatogram of CA6357 on a C18 column, using a 3-minute gradient of 5-100% acetonitrile with 0.05% formic acid. From top to bottom: total ionization chromatogram (TIC) followed by UV detection at 214, 254 and 280 nm.

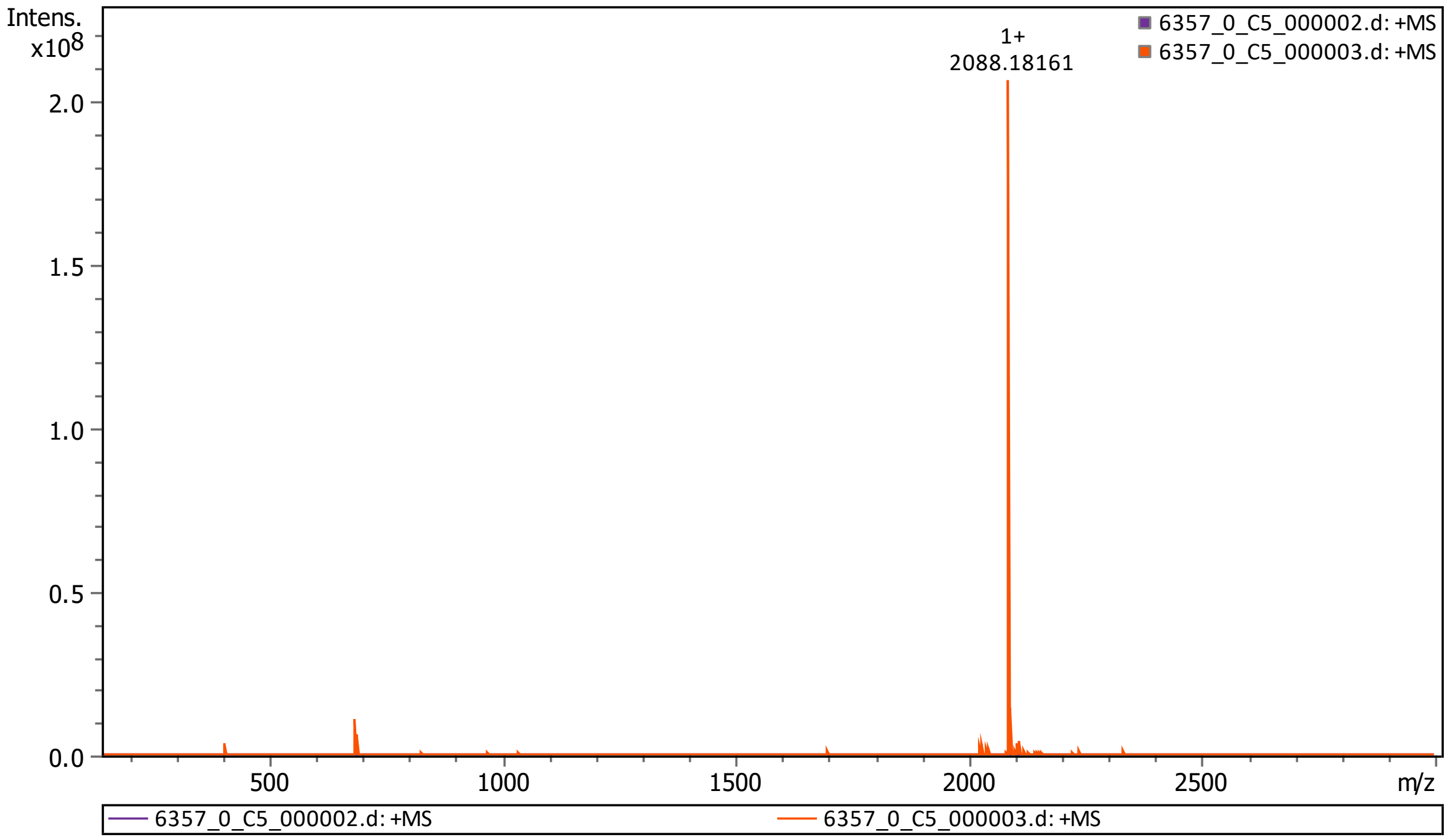


**Figure S8**. High-resolution mass spectra (HRMS) of CA6357, acquired using a matrix-assisted laser desorption/ionization (MALDI) in positive ion mode. Calculated for $C_{102}H_{156}N_{23}O_{24}$ 2088.16; found 2088.18161.

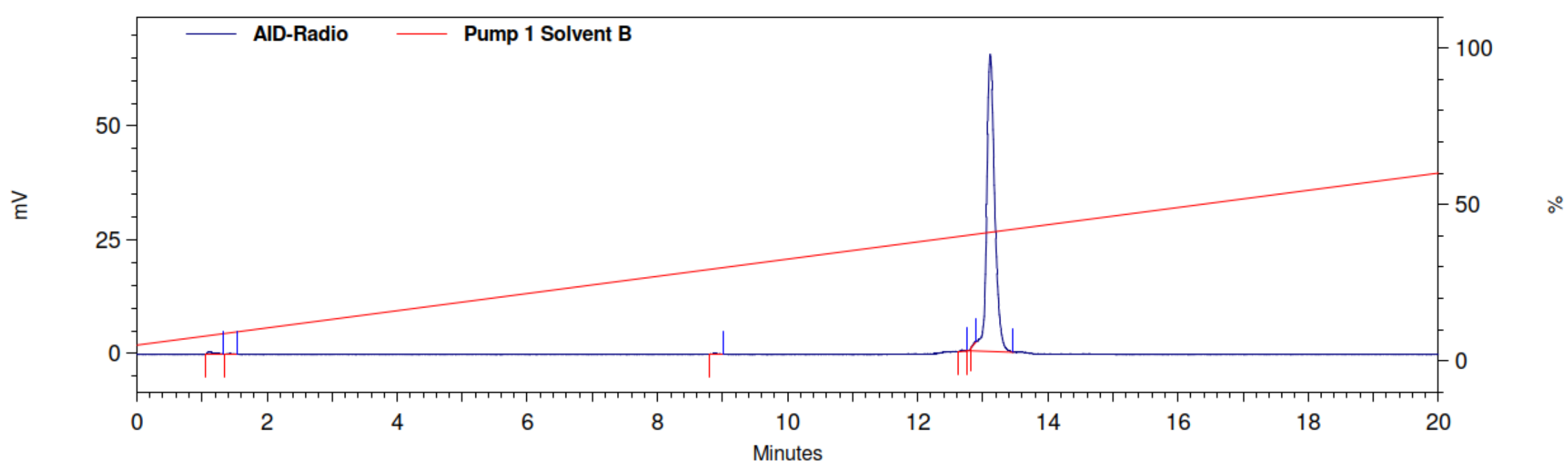


**Figure S9.** HPLC radiochromatogram of [$^{177}$Lu]Lu-CA6353 ($t_R$ 13.1 min).

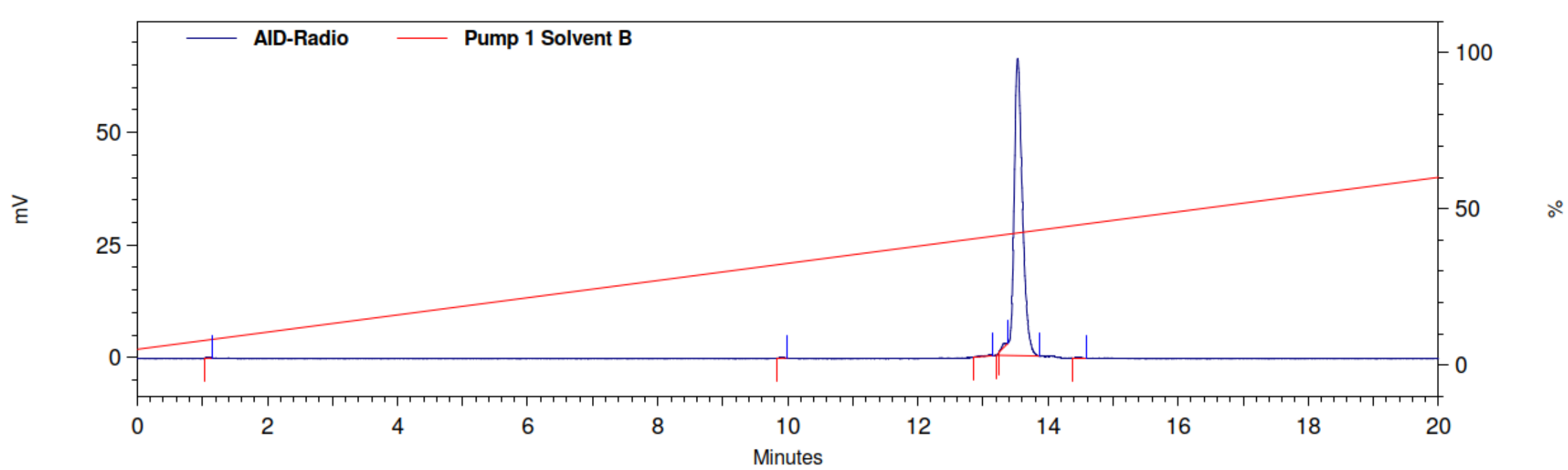


**Figure S10.** HPLC radiochromatogram of [$^{177}$Lu]Lu-CA6355 ($t_R$ 13.5 min).

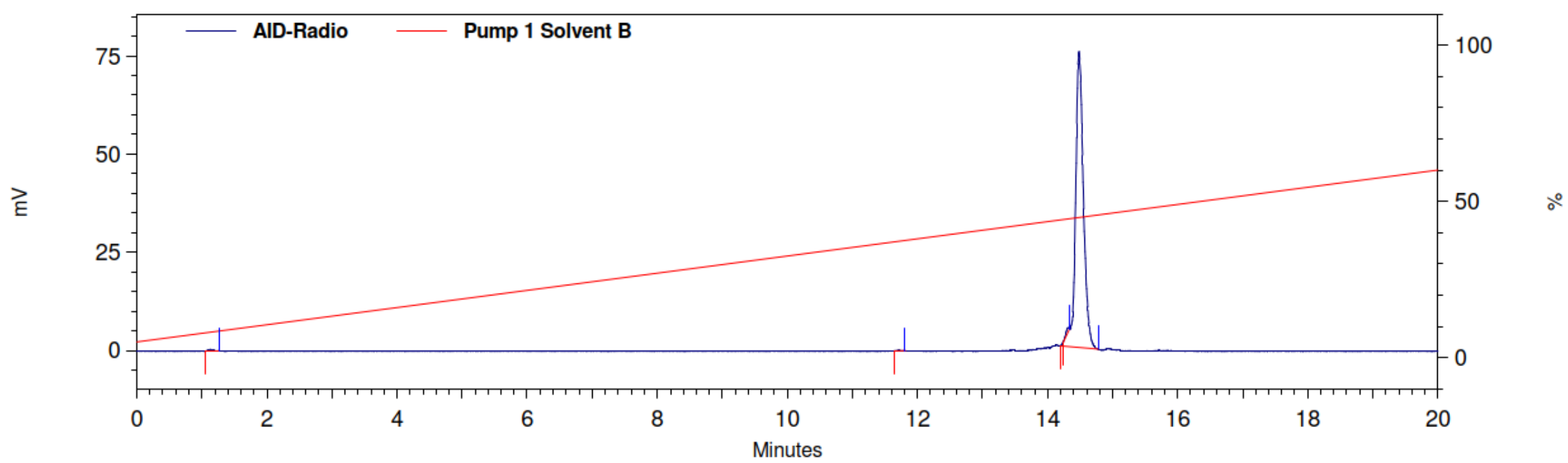


**Figure S11.** HPLC radiochromatogram of [$^{177}$Lu]Lu-CA6356 ($t_R$ 14.5 min).

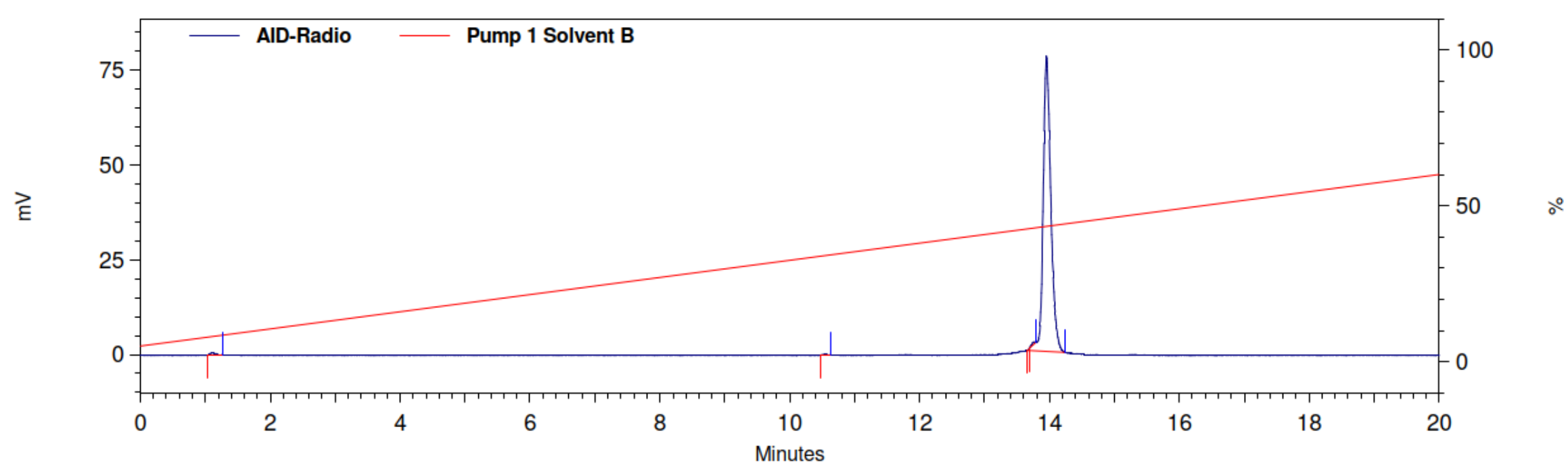


**Figure S12.** HPLC radiochromatogram of [$^{177}$Lu]Lu-CA6357 ($t_R$ 13.9 min).

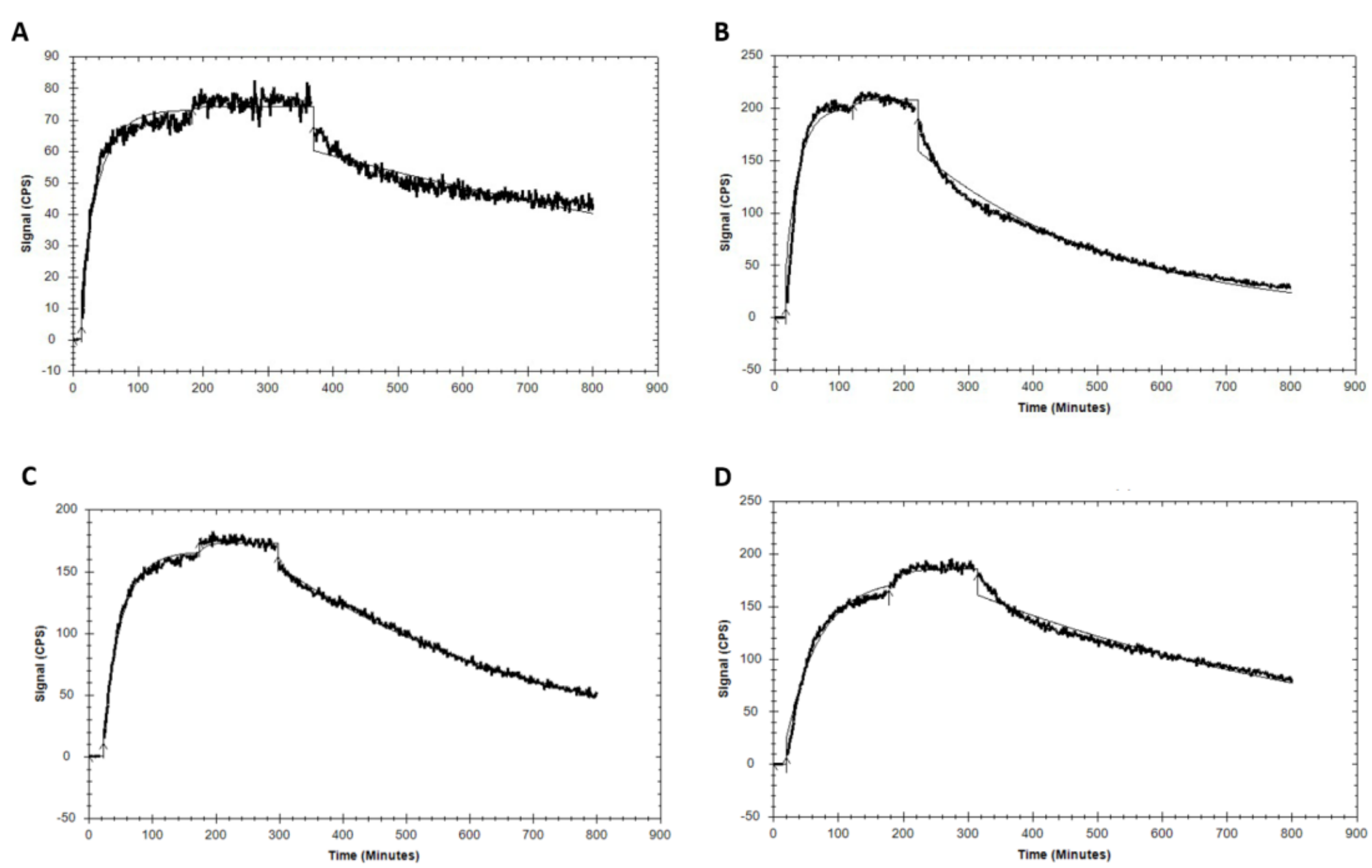


**Figure S13**. LigandTracer sensorgrams of [$^{177}$Lu]Lu-CA6353 (**A**), [$^{177}$Lu]Lu-CA6355 (**B**), [$^{177}$Lu]Lu-CA6356 (**C**), and [$^{177}$Lu]Lu-CA6357 (**D**) using a 1:1 fitting interaction mode

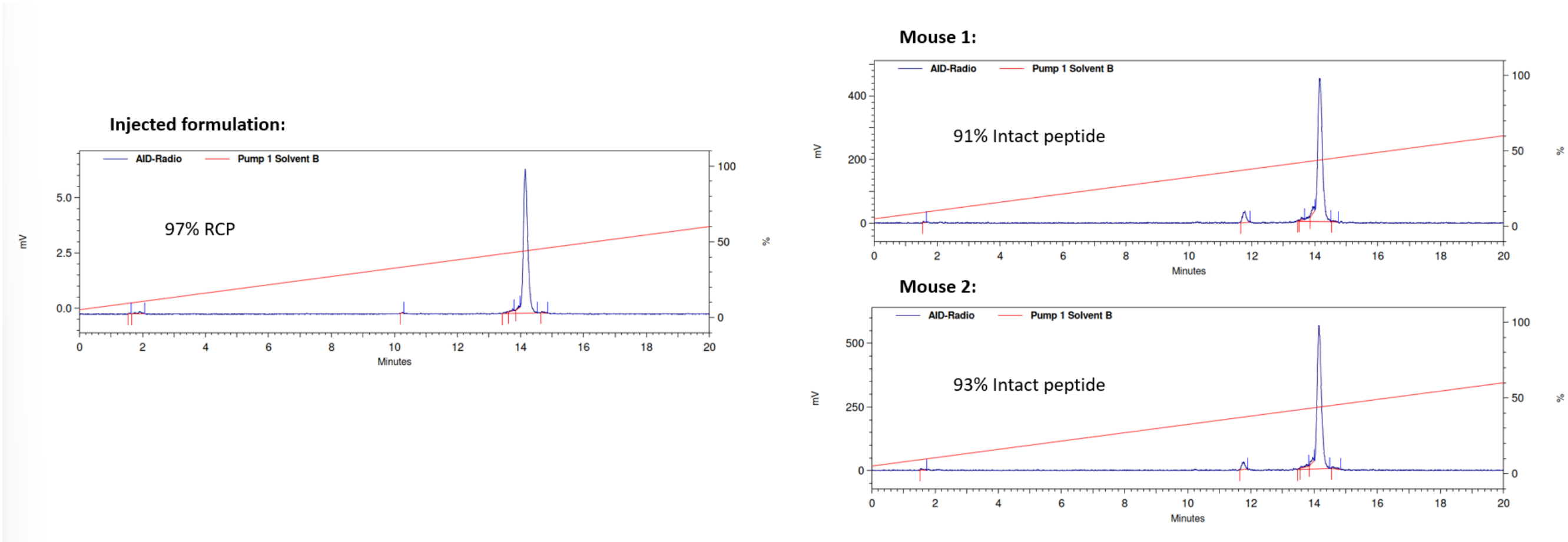


**Figure S14.** Representative radiochromatogram of the injected formulation of [$^{177}$Lu]Lu-CA6353 in NMRI mice for in vivo stability studies (RCP 97%) and of [$^{177}$Lu]Lu-CA6353 in the peripheral blood of NMRI mice 5 min pi (Mouse 1: 91% intact peptide; Mouse 2: 93% intact peptide). RCP: radiochemical purity.

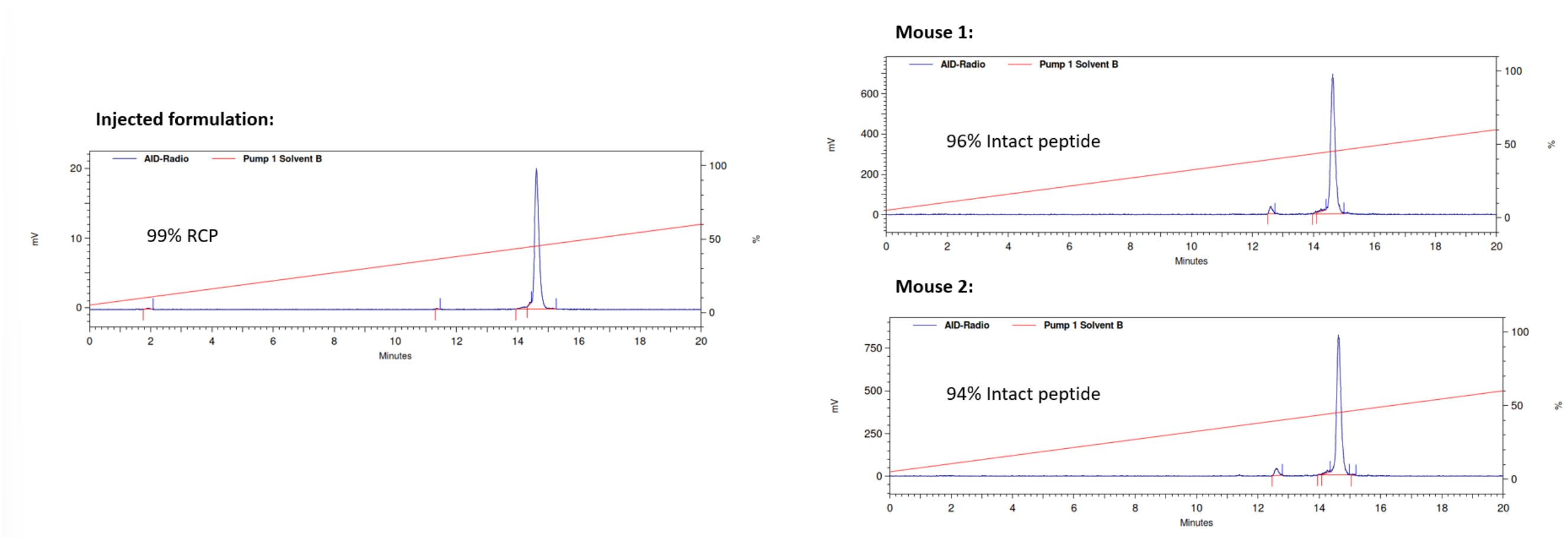


**Figure S15.** Representative radiochromatogram of the injected formulation of [$^{177}$Lu]Lu-CA6355 in NMRI mice for in vivo stability studies (RCP 99%) and of [$^{177}$Lu]Lu-CA6355 in the peripheral blood of NMRI mice 5 min pi (Mouse 1: 96% intact peptide; Mouse 2: 94% intact peptide).

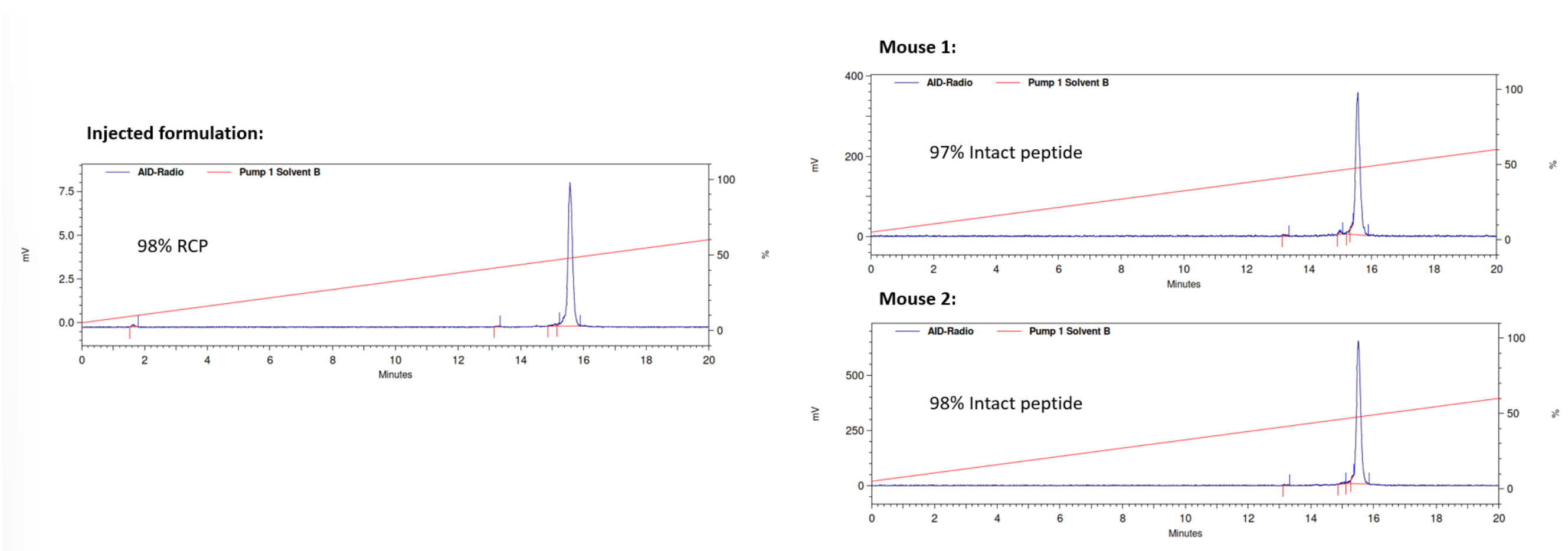


**Figure S16.** Representative radiochromatogram of the injected formulation of [$^{177}$Lu]Lu-CA6356 in NMRI mice for in vivo stability studies (RCP 98%) and of [$^{177}$Lu]Lu-CA6356 in the peripheral blood of NMRI mice 5 min pi (Mouse 1: 97% intact peptide; Mouse 2: 98% intact peptide).

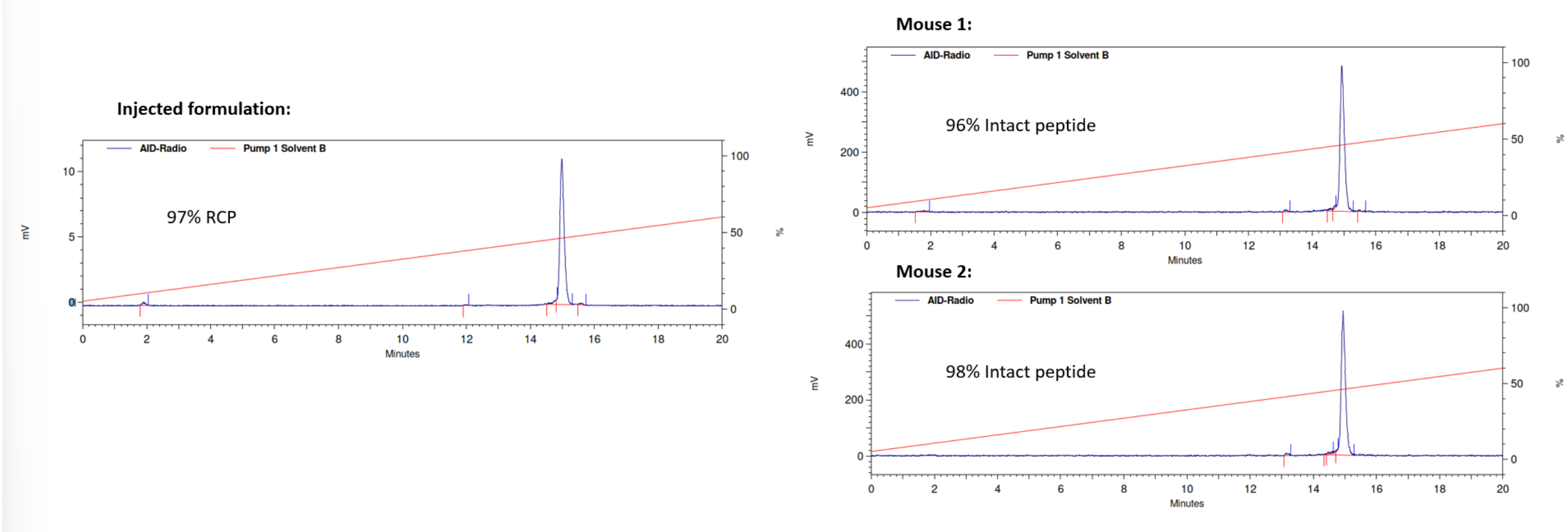


**Figure S17.** Representative radiochromatogram of the injected formulation of [$^{177}$Lu]Lu-CA6357 in NMRI mice for in vivo stability studies (RCP 97%) and of [$^{177}$Lu]Lu-CA6357 in the peripheral blood of NMRI mice 5 min pi (Mouse 1: 96% intact peptide; Mouse 2: 98% intact peptide).

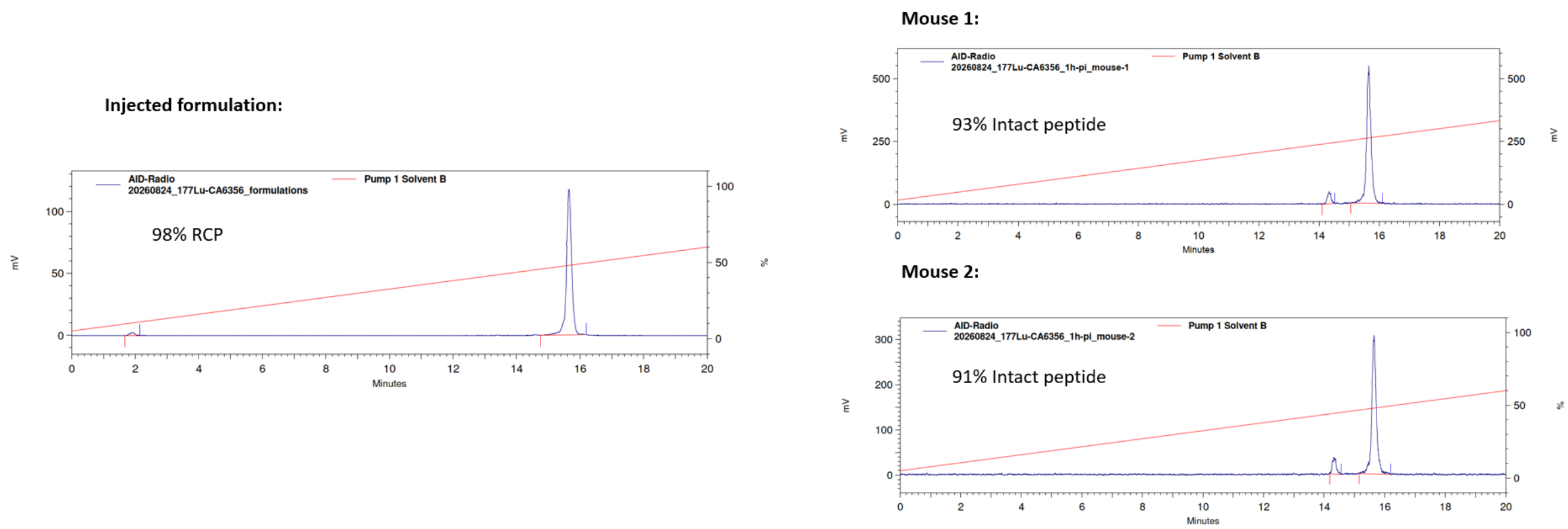


**Figure S18.** Representative radiochromatogram of the injected formulation of [$^{177}$Lu]Lu-CA6356 in NMRI mice for in vivo stability studies (RCP 98%) and of [$^{177}$Lu]Lu-CA6356 in the peripheral blood of NMRI mice at 1 h pi (Mouse 1: 93% intact peptide; Mouse 2: 91% intact peptide).

## Supplementary tables

**Table S1.** Biodistribution data (%IA/g ± SD, n=4) of [$^{177}$Lu]Lu-CA6353, [$^{177}$Lu]Lu-CA6355, [$^{177}$Lu]Lu-CA6356, [$^{177}$Lu]Lu-CA6357, and [$^{177}$Lu]Lu-PKB1 in healthy NMRI mice at 1 h and 4 h pi for [$^{177}$Lu]Lu-CA6353, [$^{177}$Lu]Lu-CA6355, [$^{177}$Lu]Lu-CA6356, [$^{177}$Lu]Lu-CA6357 and at 1 h pi for [$^{177}$Lu]Lu-PKB1. Uptake in the body is presented as %IA ± SD.

| Tissue (%IA/g) | [$^{177}$Lu]Lu-CA6353 | | [$^{177}$Lu]Lu-CA6355 | | [$^{177}$Lu]Lu-CA6356 | | [$^{177}$Lu]Lu-CA6357 | | [$^{177}$Lu]Lu-PKB1 |
|---|---|---|---|---|---|---|---|---|---|
| | **1 h pi** | **4 h pi** | **1 h pi** | **4 h pi** | **1 h pi** | **4 h pi** | **1 h pi** | **4 h pi** | **1 h pi** |
| **Blood** | 0.8 ± 0.2 | 0.05 ± 0.02 | 1.3 ± 0.3 | 0.14 ± 0.03 | 12 ± 2 | 9 ± 1 | 5 ± 1 | 1.0 ± 0.1 | 0.3 ± 0.1 |
| **Liver** | 0.9 ± 0.1 | 0.3 ± 0.1 | 1.8 ± 0.4 | 0.6 ± 0.1 | 4 ± 1 | 3.1 ± 0.2 | 3.0 ± 0.4 | 1.0 ± 0.1 | 0.21 ± 0.04 |
| **Pan-creas** | 10 ± 3 | 1.4 ± 0.3 | 15 ± 3 | 4 ± 1 | 27 ± 8 | 27 ± 4 | 27 ± 6 | 11 ± 1 | 5 ± 1 |
| **Kidneys** | 6 ± 1 | 3 ± 1 | 5 ± 1 | 3 ± 1 | 6 ± 1 | 6.3 ± 0.3 | 7 ± 1 | 5 ± 1 | 4 ± 2 |
| **Body (%IA)** | 12 ± 1 | 4 ± 2 | 18 ± 4 | 5 ± 1 | 44 ± 2 | 46 ± 5 | 34 ± 9 | 13 ± 1 | 6 ± 1 |
| Statistical differences are shown in Figure 3. | | | | | | | | | |

**Table S2.** Biodistribution data (%IA/g ± SD, n=4) of [$^{177}$Lu]Lu-PKB1, [$^{177}$Lu]Lu-CA6355, [$^{177}$Lu]Lu-CA6356, and [$^{177}$Lu]Lu-CA6357 in PC-3 xenografted mice, at 4 h pi for [$^{177}$Lu]Lu-PKB1, at 4 h and 24 h pi for [$^{177}$Lu]Lu-CA6355 and [$^{177}$Lu]Lu-CA6357, and at 4 h (blocked and non-blocked), 24 h, 72 h and 168 h pi for [$^{177}$Lu]Lu-CA6356. Uptake in the gastrointestinal tract and body is presented as %IA ± SD. NM indicates non-measurable activity.

| **Tissue (%IA/g)** | **[$^{177}$Lu]Lu-CA6355** | | **[$^{177}$Lu]Lu-CA6356** | | | | | **[$^{177}$Lu]Lu-CA6357** | | **[$^{177}$Lu]Lu-PKB1** |
|---|---|---|---|---|---|---|---|---|---|---|
| | **4 h pi** | **24 h pi** | **4 h pi (blocked)** | **4 h pi** | **24 h pi** | **72 h pi** | **168 h pi** | **4 h pi** | **24 h pi** | **4 h pi** |
| **Blood** | 0.23 ± 0.04[m] | 0.006 ± 0.004[n] | 12 ± 3 | 14 ± 2[d, e, f, g, o] | 5 ± 1[h, i, p] | 0.9 ± 0.2 | 0.03 ± 0.01 | 2.0 ± 0.2[k, l, q] | 0.11 ± 0.03 | 0.012 ± 0.001 |
| **Lungs** | 0.3 ± 0.1[m] | 0.04 ± 0.03[n] | - | 6 ± 1[d, e, f, g, o] | 3 ± 1[h, i, p] | 0.9 ± 0.2 | 0.12 ± 0.09 | 1.3 ± 0.2 | 0.23 ± 0.02 | 0.23 ± 0.17 |
| **Liver** | 1.00 ± 0.07[m] | 0.4 ± 0.1 | 4 ± 1 | 4.5 ± 0.4[d, e, f, g, o] | 1.7 ± 0.3 | 0.7 ± 0.1 | 0.3 ± 0.1 | 1.5 ± 0.1 | 0.44 ± 0.03 | 0.20 ± 0.03 |
| **Spleen** | 0.3 ± 0.1[m] | 0.1 ± 0.1[n] | - | 2.8 ± 0.4[d, f, g, o] | 2 ± 1[i, p] | 1.1 ± 0.2 | 0.5 ± 0.1 | 0.6 ± 0.1 | 0.28 ± 0.02 | 0.07 ± 0.03 |
| **Pancreas** | 6 ± 1[a, b, m] | 0.11 ± 0.04[n] | 26 ± 6[c] | 33 ± 2[d, e, f, g, o] | 6 ± 2[h, i, p] | 0.6 ± 0.1 | 0.12 ± 0.09 | 12 ± 1[k, l, q] | 0.4 ± 0.1 | 0.42 ± 0.04 |
| **Stomach** | 1.9 ± 0.4[a, b, m] | 0.12 ± 0.05[n] | 3 ± 1 | 5 ± 1[d, e, f, g, o] | 2.3 ± 0.4[h, i, p] | 0.3 ± 0.1 | NM | 3 ± 1[k, l] | 0.4 ± 0.1 | 0.4 ± 0.2 |
| **Small intestine** | 1.3 ± 0.1[m] | 0.10 ± 0.07[n] | 5 ± 2 | 7 ± 2[d, e, f, g, o] | 3 ± 1[h, i, p] | 0.2 ± 0.1 | NM | 2 ± 1[k, l] | 0.2 ± 0.1 | 0.14 ± 0.06 |
| **Kidneys** | 7 ± 1[a, b, m] | 3 ± 1[n] | 7 ± 2 | 9 ± 1[d, e, f, g, o] | 7 ± 1[h, i, p] | 3 ± 1[j] | 0.8 ± 0.3 | 7 ± 1[k, l] | 4.4 ± 0.4 | 3.2 ± 0.2 |
| **Tumour** | 10 ± 1[a, b] | 7 ± 2[n] | 5 ± 3[c] | 12 ± 2[d, e, f, g] | 15 ± 2[h, i, p] | 4 ± 1[j] | 1.4 ± 0.3 | 12 ± 1[k, l, q] | 8 ± 1[r] | 7 ± 1 |
| **Muscle** | 0.09 ± 0.02 | 0.02 ± 0.01 | - | 1.4 ± 0.3 | 0.7 ± 0.1 | 0.2 ± 0.1 | 0.02 ± 0.03 | 0.26 ± 0.04 | 0.05 ± 0.01 | 0.016 ± 0.003 |
| **Bone** | 0.3 ± 0.1 | 0.12 ± 0.06 | - | 1.8 ± 0.2[f, g] | 1.2 ± 0.2 | 0.3 ± 0.1 | NM | 0.4 ± 0.1 | 0.2 ± 0.1 | 0.05 ± 0.01 |
| **Gastrointestinal tract (%IA)** | 3 ± 1[a, b, m] | 0.22 ± 0.03[n] | - | 7 ± 1[d, e, f, g, o] | 2 ± 1[h, i, p] | 0.6 ± 0.1 | 0.18 ± 0.03 | 4 ± 1[k, l] | 0.4 ± 0.1 | 0.7 ± 0.1 |
| **Body (%IA)** | 4 ± 1[a, b, m] | 1.0 ± 0.3[n] | 33 ± 6[c] | 27 ± 2[d, e, f, g, o] | 14 ± 2[h, i, p] | 6 ± 1[j] | 2.1 ± 0.3 | 6 ± 1[k, l, q] | 1.5 ± 0.2 | 0.9 ± 0.5 |

[a-r] Two-way Anova with Šidák post hoc analysis ($p < 0.05$): [a] Statistical difference between [$^{177}$Lu]Lu-CA6355 and [$^{177}$Lu]Lu-PKB1 at 4 h pi;
[b] Statistical difference between [$^{177}$Lu]Lu-CA6355 at 4 h and 24 h pi; [c] Statistical difference between [$^{177}$Lu]Lu-CA6356 at 4 h pi blocked vs non blocked; [d] Statistical difference between [$^{177}$Lu]Lu-CA6356 and [$^{177}$Lu]Lu-PKB1 at 4 h pi; [e] Statistical difference between [$^{177}$Lu]Lu-CA6356 at 4 h and 24 h pi; [f] Statistical difference between [$^{177}$Lu]Lu-CA6356 4 h and 72 h pi; [g] Statistical difference between [$^{177}$Lu]Lu-CA6356 4 h and 168 h pi; [h] Statistical difference between [$^{177}$Lu]Lu-CA6356 24 h and 72 h pi; [i] Statistical difference between [$^{177}$Lu]Lu-CA6356 24 h and 168 h pi; [j] Statistical difference between [$^{177}$Lu]Lu-CA6356 72 h and 168 h pi; [k] Statistical difference between [$^{177}$Lu]Lu-CA6357 and [$^{177}$Lu]Lu-PKB1 at 4 h pi; [l] Statistical difference between [$^{177}$Lu]Lu-CA6357 at 4 h and 24 h pi; [m] Statistical difference between [$^{177}$Lu]Lu-CA6355 and [$^{177}$Lu]Lu-CA6356 at 4 h pi; [n] Statistical difference between [$^{177}$Lu]Lu-CA6355 and [$^{177}$Lu]Lu-CA6356 at 24 h pi; [o] Statistical difference between [$^{177}$Lu]Lu-CA6356 and [$^{177}$Lu]Lu-CA6357 at 4 h pi; [p] Statistical difference between

$[^{177}Lu]$Lu-CA6356 and $[^{177}Lu]$Lu-CA6357 at 24 h pi; [q] Statistical difference between $[^{177}Lu]$Lu-CA6355 and $[^{177}Lu]$Lu-CA6357 at 4 h pi; [r] Statistical difference between $[^{177}Lu]$Lu-CA6355 and $[^{177}Lu]$Lu-CA6357 at 24 h pi;

## References

1. (a) Wagh, NK, Zhou Z, Ogbomo SM, Shi W, Brusnahan SK, Garrison JC. Development of Hypoxia Enhanced 111In-Labeled Bombesin Conjugates: Design, Synthesis, and In Vitro Evaluation in PC-3 Human Prostate Cancer. Bioconjugate Chem. 2012;23 (3): 527–537. (b) Pagoto, A., Garello, F., Marini, G.M. et al. Novel Gastrin-Releasing Peptide Receptor Targeted Near-Infrared Fluorescence Dye for Image-Guided Surgery of Prostate Cancer. Mol Imaging Biol. 2020;22:85–93. (c) Varasteh Z, Velikyan I, Lindeberg G, Sörensen J, Larhed M, Sandström M, et. al. Synthesis and Characterization of a High-Affinity NOTA-Conjugated Bombesin Antagonist for GRPR-Targeted Tumor Imaging. Bioconjugate Chem. 2013;24(7):1144–1153.

2. Bjäreback A, Jonmarker O, Tzortzakakis A, Jussing E, Li C, Altena R, et al. First-in-human experience with GRPR antagonist [68Ga]Ga-NOTA-PEG2-RM26 in prostate and breast cancer patients using PET/CT. EJNMMI Res. 2025;15(1):12
